# Supplementary material for: Delivery of oligonucleotides to bone marrow to modulate ferrochelatase splicing in a mouse model of erythropoietic protoporphyria
Source: Nucleic Acids Res. 2020 Apr 20;48(9):4658–71. doi: 10.1093/nar/gkaa229 (PMC7229840; doi:10.1093/nar/gkaa229)
Supplement: gkaa229_Supplemental_File [file gkaa229_supplemental_file.pdf]

Supplementary information

Delivery of oligonucleotides to bone marrow to modulate ferrochelatase splicing in a mouse model of Erythropoietic Protoporphyria

François Halloy[1], Pavithra S. Iyer[1], Paulina Ćwiek[1], Alice Ghidini[1], Jasmin Barman-Aksözen[2], Nicole Wildner-Verhey van Wijk[3], Alexandre Theocharides[3], Elisabeth I. Minder[4], Xiaoye Schneider-Yin[2], Daniel Schümperli[1*] and Jonathan Hall[1*]

[1]Institute of Pharmaceutical Sciences, Department of Chemistry and Applied Biosciences, ETH Zurich, Switzerland
[2]Institute of Laboratory Medicine, Municipal Hospital Triemli, Zurich, Switzerland
[3]Department of Medical Oncology and Hematology, University Hospital and University of Zurich, Switzerland
[4]Department of Internal Medicine, Municipal Hospital Triemli, Zurich, Switzerland
Correspondence: daniel.schuemperli@pharma.ethz.ch and jonathan.hall@pharma.ethz.ch

Table of Contents

Supplementary figures 3

Figure S1. Oligonucleotide analytics for *in vitro* work in COS-7 and K562 cells 3

Figure S2. Oligonucleotide analytics of CL-qPCR primers 11

Figure S3. Analytics of in-vivo batches of ORN1, ORN_{st}, ORN_{84} and ORN_{ch} 13

Figure S4. Gel replicates for *FECH* endogenous splicing correction in K562 cells. 15

Figure S5. Western Blot replicates for FECH endogenous splicing correction in K562 cells. .. 16

Figure S6. Aberrant splicing generated by the *FECH* c.315-48T and C minigenes 17

Figure S7. Status of the c.315-48 polymorphism in K562 cells*:* alignment of K562 gDNA sequencing data with *FECH* Human Reference Sequence (GRCh38.p13) 17

Figure S8. Calibration curves of oligonucleotide conjugates in kidney lysate 18

In vivo metabolism study in mouse tissues: mass spectrometry data (Figures S9 to S12) 19

Figure S9. MS data of ORN1 in tissues 19

Figure S10. MS data of ORN_{st} in tissues 22

Figure S11. MS data of ORN_{84} in tissues 24

Figure S12. MS data of ORN_{ch} in tissues 27

Figure S13. Splicing correction generated by ORN1, ORN_{st}, ORN_{ch} and ORN_{84} in liver of *Emi/wt* mice. 31

Supplementary tables 32

Table S1. Library of 10-nt ORNs for minigene screening 32

Supplementary methods 33

Deprotection and purification conditions for lead oligonucleotide ORN1 33

Conjugation on support, deprotection and purification of stearic acid conjugate ORN_{st} 33

Deprotection of maleimide-protected form of ORN1 and retro Diels Alder uncaging for conjugation to thiol ligands 33

Thiocholesteryl conjugate preparation ORN_{ch} from the uncaged maleimide intermediate 34

References 34

Supplementary figures

Figure S1. Oligonucleotide analytics for *in vitro* work in COS-7 and K562 cells

Samples were analysed by LC-MS (Agilent 1200/6130 system) equipped with a Waters Acquity OST C-18 column with a gradient of MeOH in 0.4 mM HFIP, 15 mM trimethylamine; flow rate: 0.3 ml/min. The target refers to the position bound on *FECH* pre-mRNA.

Name	Seq. 5'-3'	Target	Chemistry	MW found (g/mol)	MW calc. (g/mol)	Δm (%)	UV purity (%)
**1**	g^{m}c^{m}ctaa^{m}caagattaag^{m}c^{m}ct	-102-120	MOE-PS	7547.22	7547	0.01	98

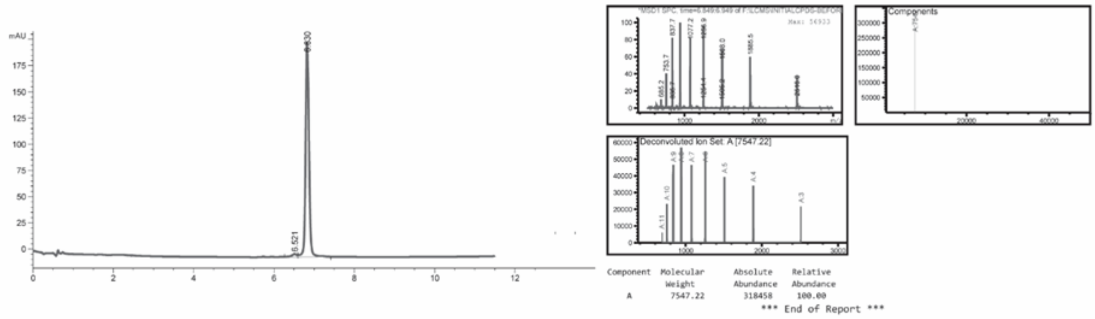

Name	Seq. 5'-3'	Target	Chemistry	MW found (g/mol)	MW calc. (g/mol)	Δm (%)	UV purity (%)
**2**	tttagagag^{m}c^{m}ctaa^{m}caaga	-94-112	MOE-PS	7582.98	7582	0.01	>99

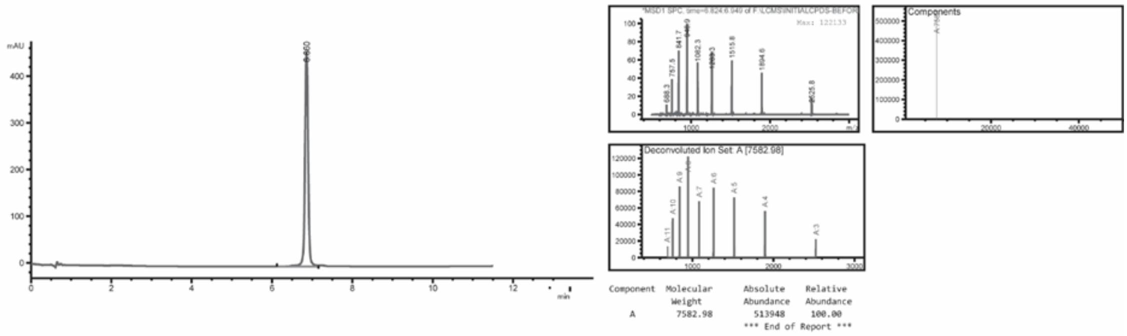

Name	Seq. 5'-3'	Target	Chemistry	MW found (g/mol)	MW calc. (g/mol)	Δm (%)	UV purity (%)
**3**	ag^{m}caaaattttagagag^{m}c^{m}c	-86-104	MOE-PS	7582.45	7582	0.01	>99

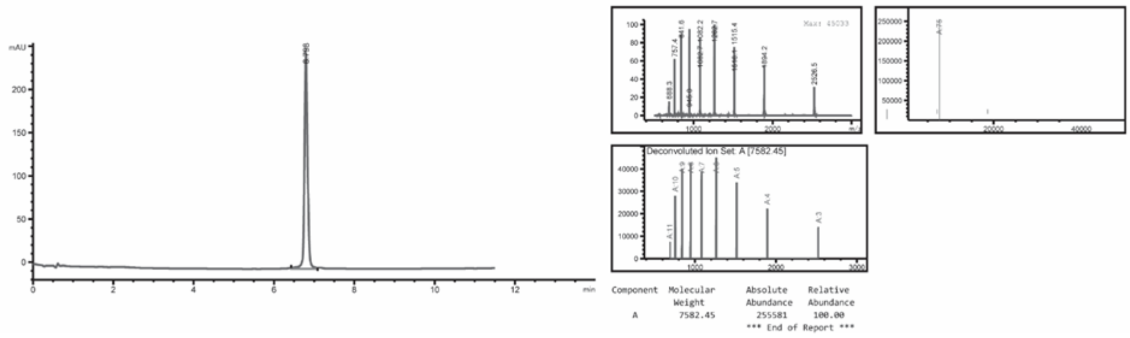

Name	Seq. 5'-3'	Target	Chemistry	MW found (g/mol)	MW calc. (g/mol)	Δm (%)	UV purity (%)
**4**	agaaaaaaag^{m}caaaatttt	-78-96	MOE-PS	7573.13	7571	0.02	98

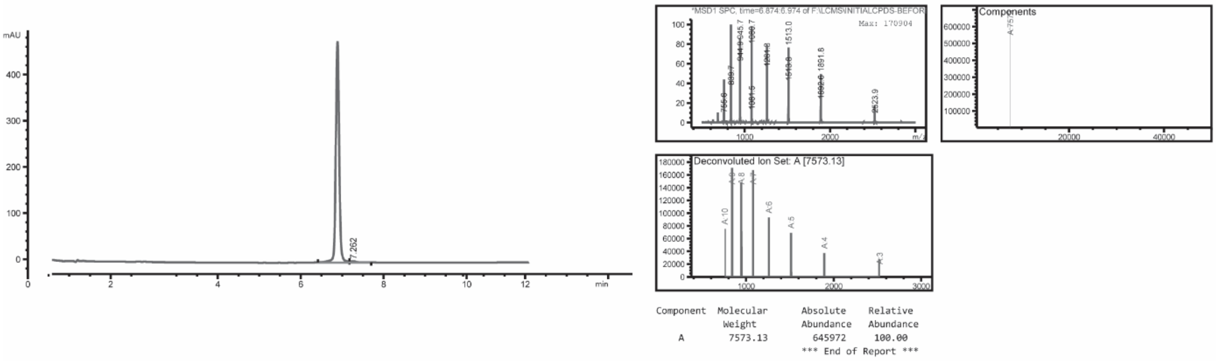

Name	Seq. 5'-3'	Target	Chemistry	MW found (g/mol)	MW calc. (g/mol)	Δm (%)	UV purity (%)
**5**	aataaaaaagaaaaaaag^{m}c	-70-88	MOE-PS	7598.62	7597	0.02	>99

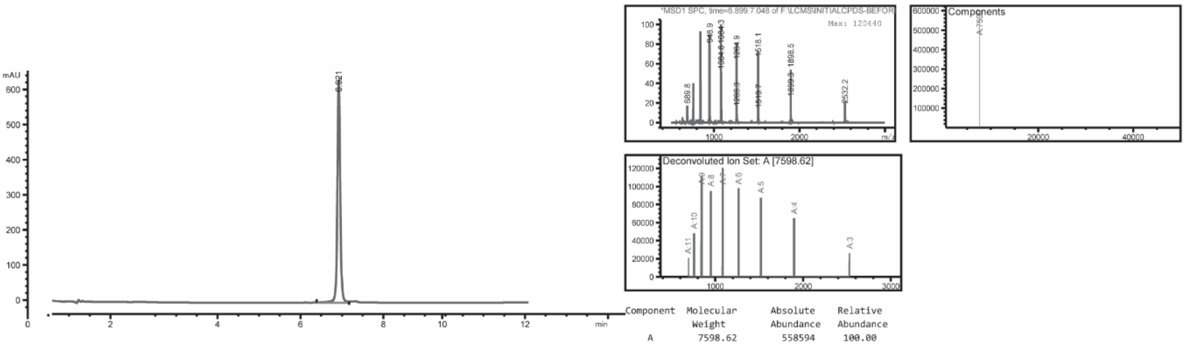

Name	Seq. 5'-3'	Target	Chemistry	MW found (g/mol)	MW calc. (g/mol)	Δm (%)	UV purity (%)
**6**	tt^{m}cta^{m}ctcaauaaaaaaga	-62-80	MOE-PS	7525.52	7525	0.01	>99

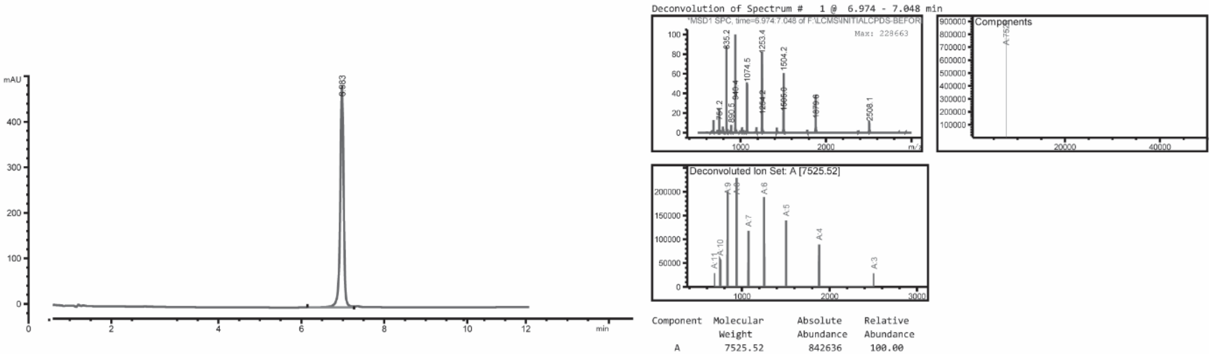

Name	Seq. 5'-3'	Target	Chemistry	MW found (g/mol)	MW calc. (g/mol)	Δm (%)	UV purity (%)
**7**	Gaaatgttttctactcaat	-54-72	MOE-PS	7515.80	7514	0.01	>99

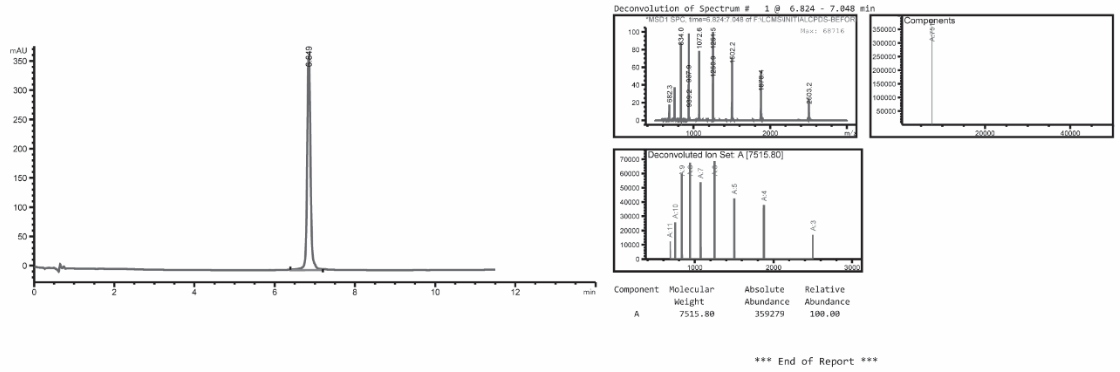

Name	Seq. 5'-3'	Target	Chemistry	MW found (g/mol)	MW calc. (g/mol)	Δm (%)	UV purity (%)
**8**	gagaaatgtttt^{m}cta^{m}ct^{m}ca	-52-70	MOE-PS	7555.33	7553	0.01	>99

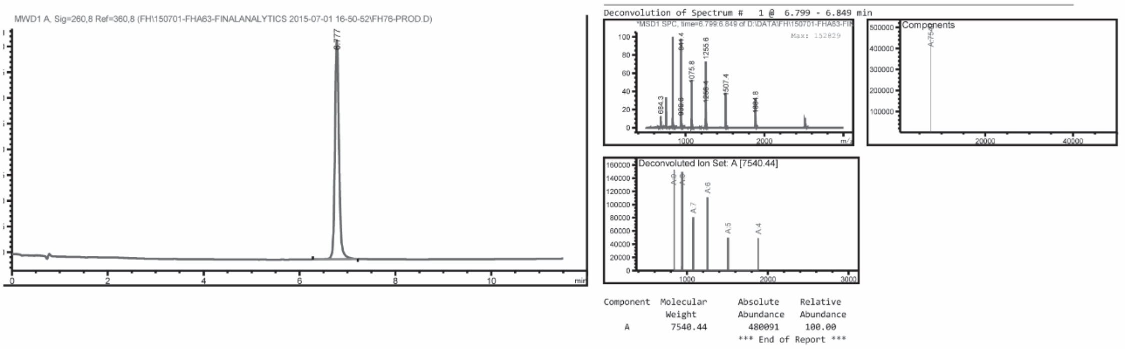

Name	Seq. 5'-3'	Target	Chemistry	MW found (g/mol)	MW calc. (g/mol)	Δm (%)	UV purity (%)
**9**	tgagaaatgtttt^{m}cta^{m}ct^{m}c	-51-69	Full MOE-PS	7530.69	7529.8	0.01	>99

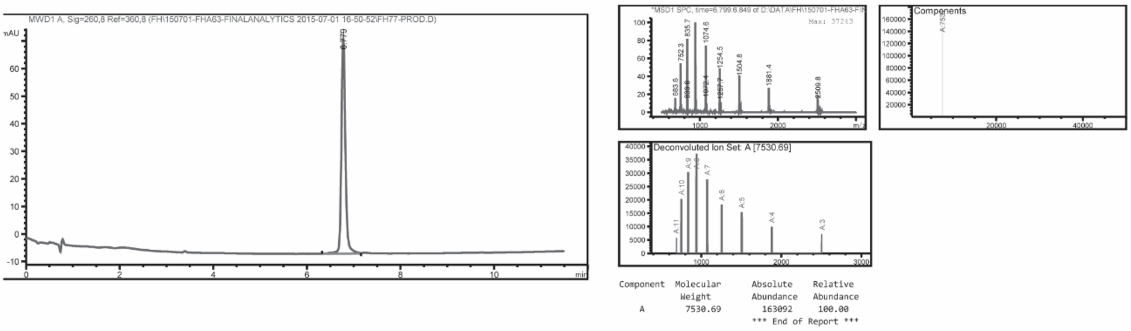

Name	Seq. 5'-3'	Target	Chemistry	MW found (g/mol)	MW calc. (g/mol)	Δm (%)	UV purity (%)
**10**	^{m}ctgagaaatgtttt^{m}cta^{m}ct	-50-68	Full MOE-PS	7530.69	7529.8	0.01	>99

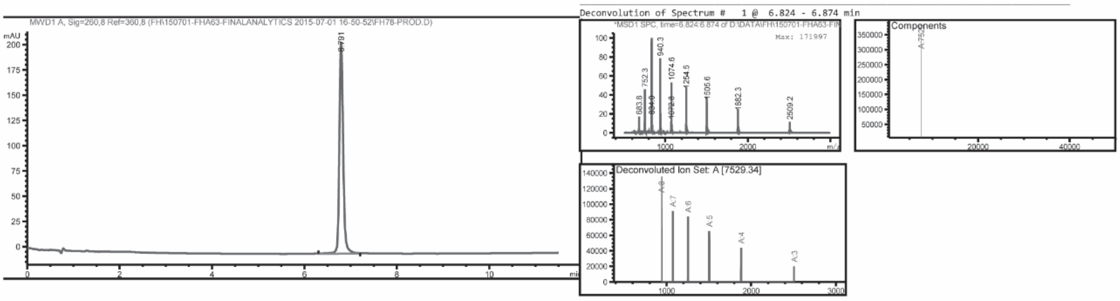

Component	Molecular Weight	Absolute Abundance	Relative Abundance
A	7529.34	357015	100.00

*** End of Report ***

Name	Seq. 5'-3'	Target	Chemistry	MW found (g/mol)	MW calc. (g/mol)	Δm (%)	UV purity (%)
**11**	^{m}c^{m}ctgagaaatgtttt^{m}cta^{m}c	-49-67	Full MOE-PS	7531.24	7528.8	0.03	>99

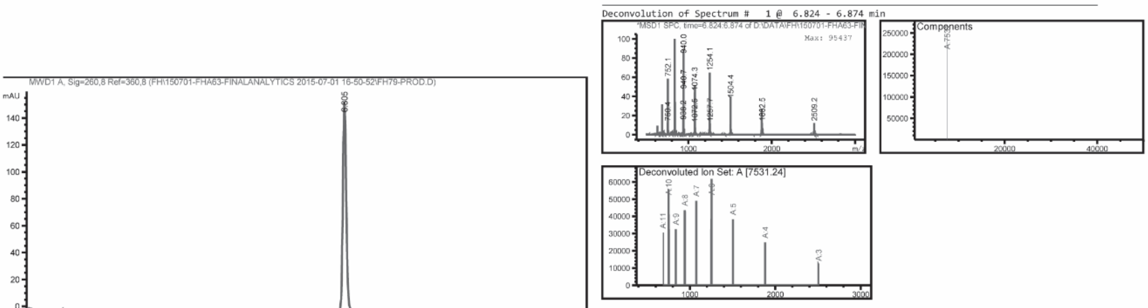

Component	Molecular Weight	Absolute Abundance	Relative Abundance
A	7531.24	249348	100.00

*** End of Report ***

Name	Seq. 5'-3'	Target	Chemistry	MW found (g/mol)	MW calc. (g/mol)	Δm (%)	UV purity (%)
**12**	g^{m}c^{m}ctgagaaatgtttt^{m}cta	-48-66	Full MOE-PS	7555.40	7554.8	0.01	>99

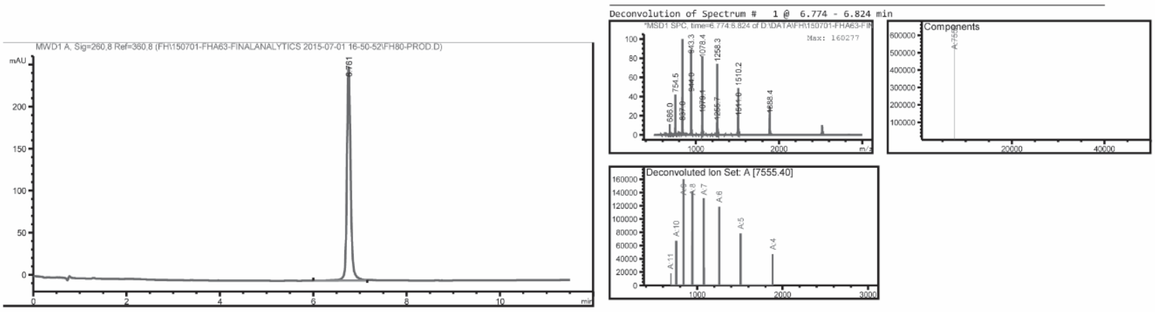

Component	Molecular Weight	Absolute Abundance	Relative Abundance
A	7555.40	610300	100.00

*** End of Report ***

Name	Seq. 5'-3'	Target	Chemistry	MW found (g/mol)	MW calc. (g/mol)	Δm (%)	UV purity (%)
**13**	ag^{m}c^{m}ctgagaaatgtttt^{m}ct	-47-65	MOE-PS	7556.59	7555	0.01	>99

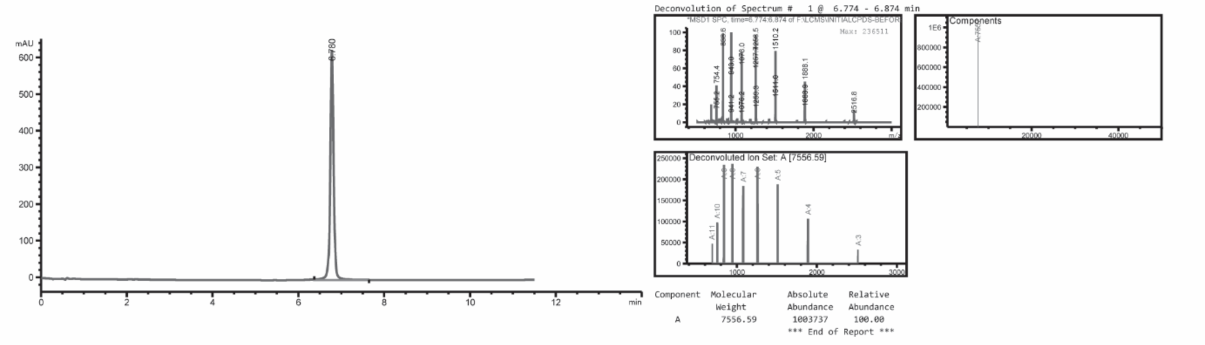

Name	Seq. 5'-3'	Target	Chemistry	MW found (g/mol)	MW calc. (g/mol)	Δm (%)	UV purity (%)
**14**	^{m}cag^{m}c^{m}ctgagaaatgtttt^{m}c	-46-64	MOE-PS	7555.33	7553	0.01	>99

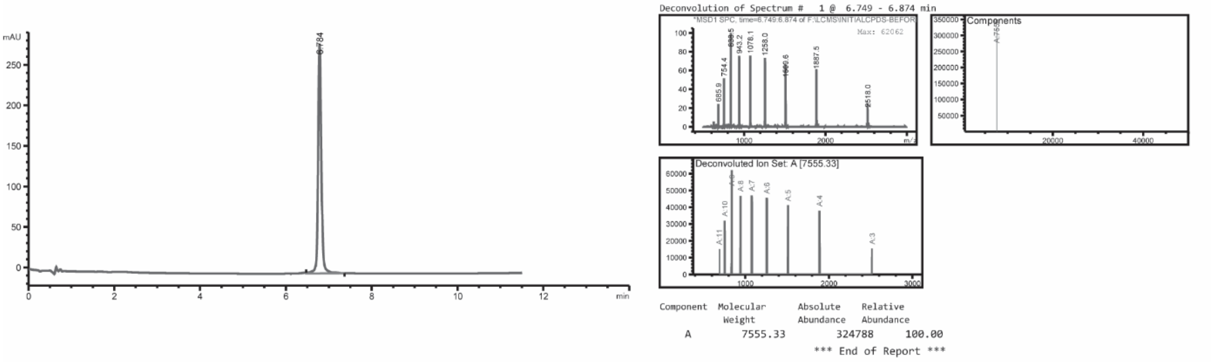

Name	Seq. 5'-3'	Target	Chemistry	MW found (g/mol)	MW calc. (g/mol)	Δm (%)	UV purity (%)
**15**	g^{m}cag^{m}c^{m}ctgagaaatgtttt	-45-63	MOE-PS	7580.48	7580	0.01	>99

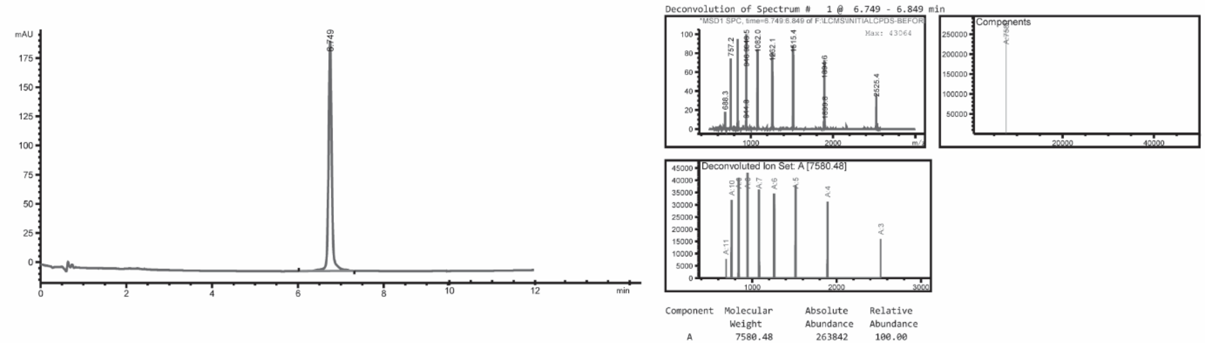

Name	Seq. 5'-3'	Target	Chemistry	MW found (g/mol)	MW calc. (g/mol)	Δm (%)	UV purity (%)
**16**	ag^{m}cag^{m}c^{m}ctgagaaatgttt	-44-62	Full MOE-PS	7588.02	7588.9	0.01	>99

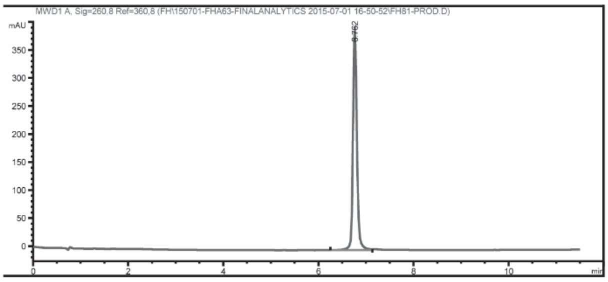

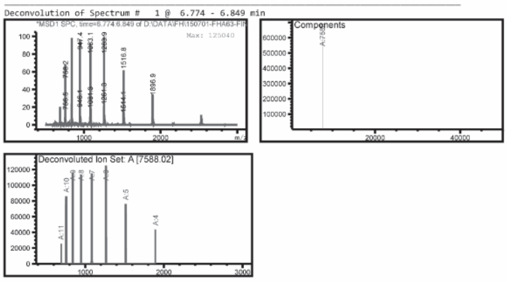

Name	Seq. 5'-3'	Target	Chemistry	MW found (g/mol)	MW calc. (g/mol)	Δm (%)	UV purity (%)
**17**	tag^{m}cag^{m}c^{m}ctgagaaatgtt	-43-61	Full MOE-PS	7589.52	7588.9	0.01	>99

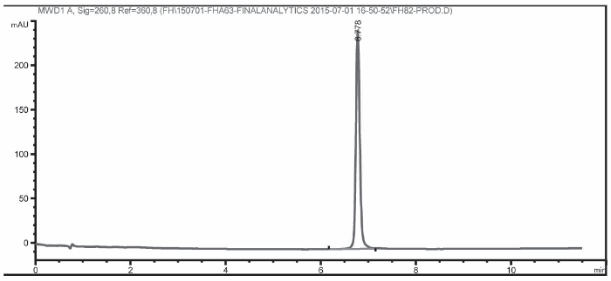

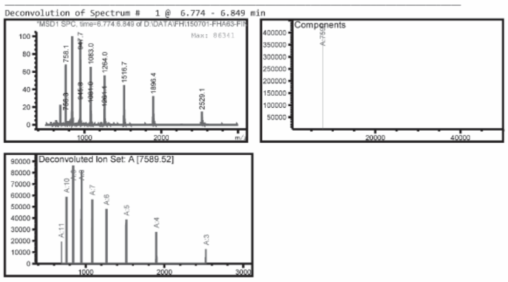

Name	Seq. 5'-3'	Target	Chemistry	MW found (g/mol)	MW calc. (g/mol)	Δm (%)	UV purity (%)
**18**	ttag^{m}cag^{m}c^{m}ctgagaaatgt	-42-60	Full MOE-PS	7589.09	7588.9	<0.01	>99

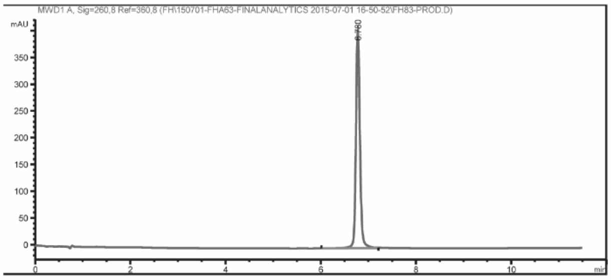

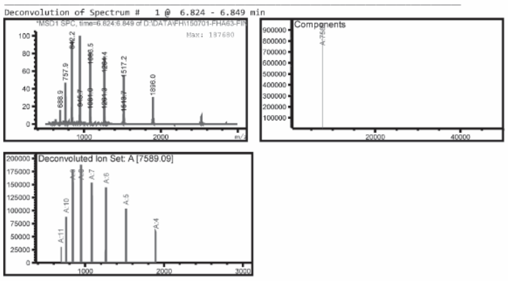

Name	Seq. 5'-3'	Target	Chemistry	MW found (g/mol)	MW calc. (g/mol)	Δm (%)	UV purity (%)
**19**	^{m}cttag^{m}cag^{m}c^{m}ctgagaaatg	-41-59	Full MOE-PS	7588.54	7587.9	0.01	98.7

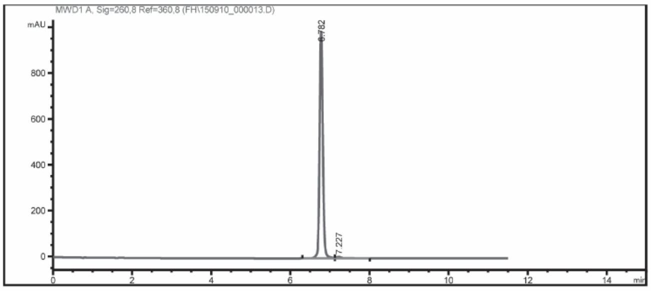

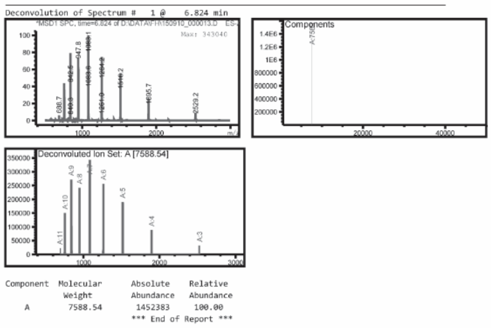

Name	Seq. 5'-3'	Target	Chemistry	MW found (g/mol)	MW calc. (g/mol)	Δm (%)	UV purity (%)
**20**	g^{m}cttag^{m}cag^{m}c^{m}ctgagaaat	-40-58	Full MOE-PS	7588.83	7587.9	0.01	>99

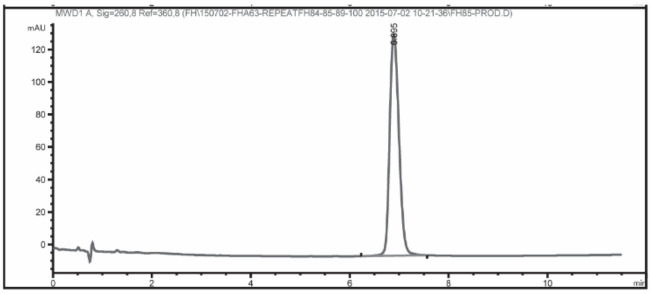

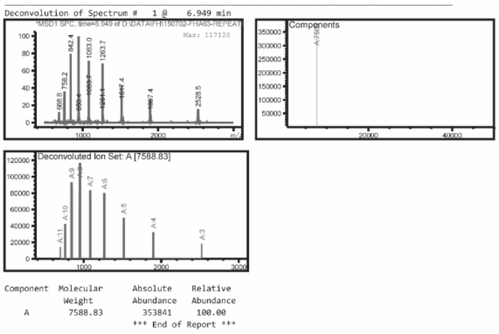

Neg Ctrl 1

Name	Seq. 5'-3'	Target	Chemistry	MW found (g/mol)	MW calc. (g/mol)	Δm (%)	UV purity (%)
**21**	gttattg^{m}ctat^{m}cgaag^{m}cag	none	Full MOE-PS	7583.00	7579.9	0.04	>99

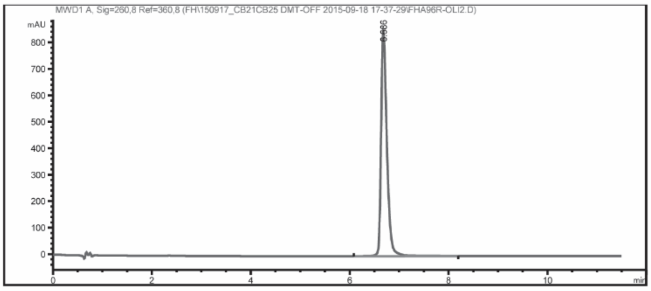

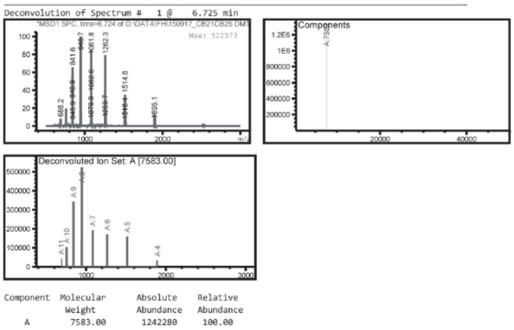

22 : LNA/DNA mixmer of sequence -45-63. Synthesized at Exiqon (now Qiagen).

ORN1

Name	Seq. 5'-3'	Target	Chemistry	MW found (g/mol)	MW calc. (g/mol)	Δm (%)	UV purity (%)
**23**	ag^{m}cag^{m}c^{m}ctgagaaatgtttt^{m}ct	-44-65	Full MOE-PS	8772.96	8770.6	0.03	98.4

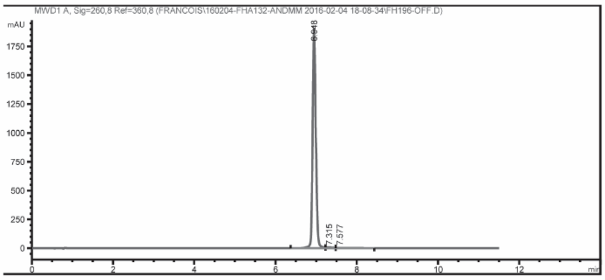

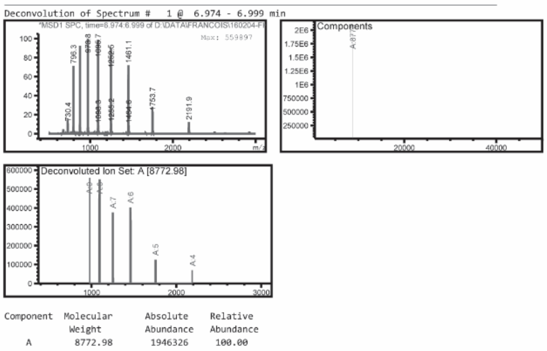

ORN2

Name	Seq. 5'-3'	Target	Chemistry	MW found (g/mol)	MW calc. (g/mol)	Δm (%)	UV purity (%)
**24**	^{m}cttag^{m}cag^{m}c^{m}ctgagaaatgttt	-41-62	Full MOE-PS	8771.37	8770.6	0.01	98.6

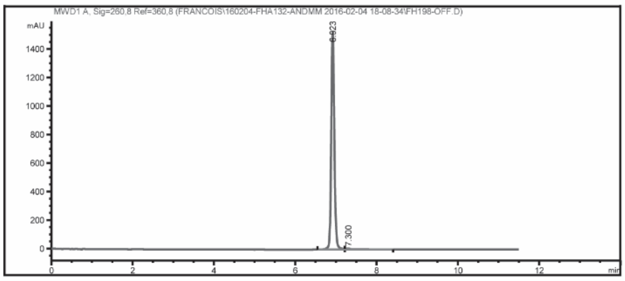

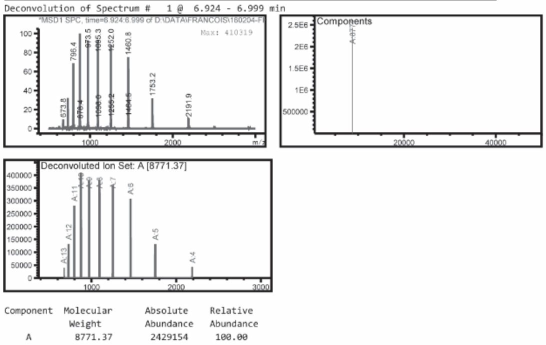

ORN3

Name	Seq. 5'-3'	Target	Chemistry	MW found (g/mol)	MW calc. (g/mol)	Δm (%)	UV purity (%)
**25**	^{m}cttag^{m}cag^{m}c^{m}ctgagaaatgtttt^{m}ct	-41-65	Full MOE-PS	9953.08	9952.4	0.01	99.6

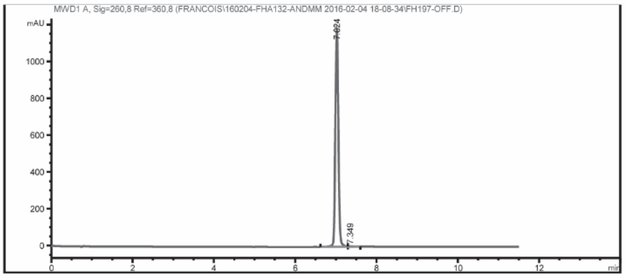

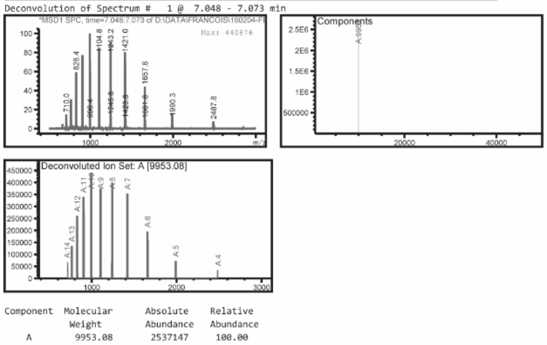

	Name	Seq. 5'-3'	Target	Chemistry	MW found (g/mol)	MW calc. (g/mol)	∆m (%)	UV purity (%)
**Neg Ctrl 2**	**26**	gt^{m}cgttagaa^{m}ctta^{m}ctgga^{m}cta	none	Full MOE-PS	8771.42	8770.6	0.01	>99

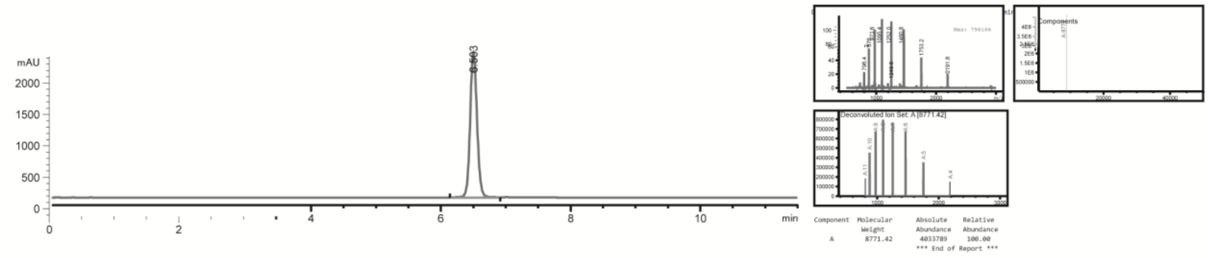

	Name	Seq. 5'-3'	Target	Chemistry	MW found (g/mol)	MW calc. (g/mol)	∆m (%)	UV purity (%)
**Neg Ctrl 3**	**27**	ata^{m}cg^{m}cgtattata^{m}cg^{m}cgattaa^{m}cga^{m}c	none	Full MOE-PS	10768.1	10767	0.01	94

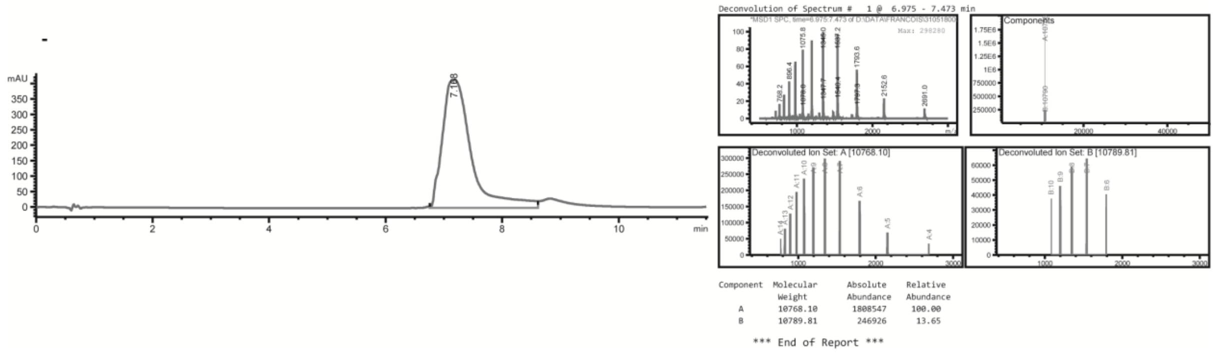

Figure S2. Oligonucleotide analytics of CL-qPCR primers

Samples were analysed by LC-MS using conditions described above. Cy3 azide and BHQ azide moieties were coupled on oligonucleotide sequences with 2'-*O*-propargyl modifications according to an in-house protocol (1).

Name	Seq. 5'-3'	Chemistry	MW found (g/mol)	MW calc. (g/mol)	∆m (%)	UV purity (%)
**28**	TTAAACCAAGAAAACATTT$_{PS}$	DNA-PO + terminal PS	5883.13	5883.94	0.01	97.5

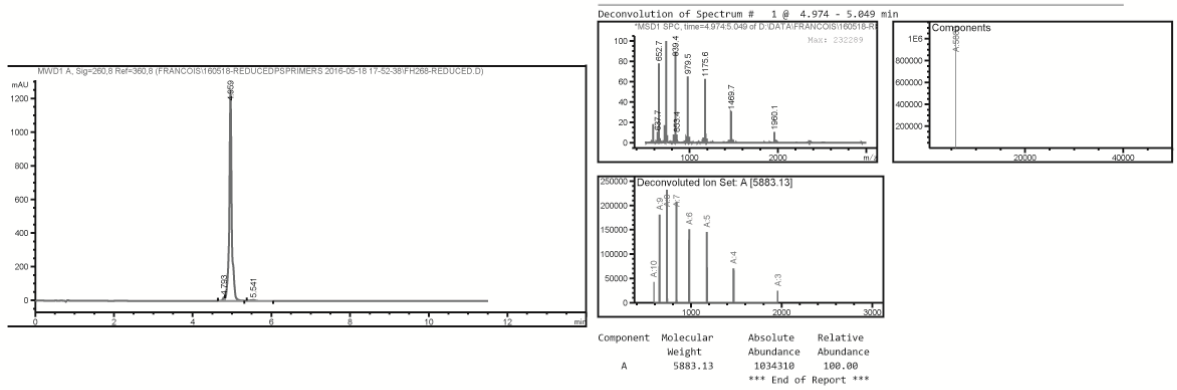

Name	Seq. 5'-3'	Chemistry	MW found (g/mol)	MW calc. (g/mol)	Δm (%)	UV purity (%)
**29**	ACGTGGTTAGCAGCCTGAGAA	DNA-PO	6494.9, Na salt	6495.3	0.01	>99

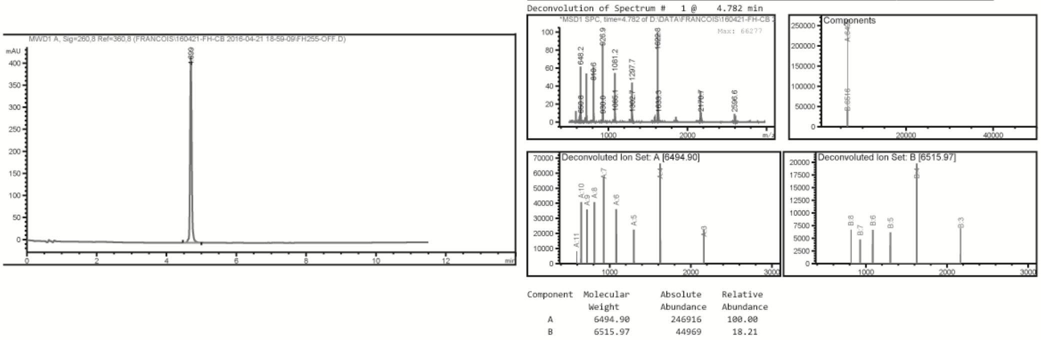

Name	Seq. 5'-3'	Chemistry	MW found (g/mol)	MW calc. (g/mol)	Δm (%)	UV purity (%)
**30**	C$_{2'\text{-O-Cy3}}$TCCCTCCCTCGATTTAAACCAAGAAAACAT	DNA-PO Cy3 label	9967.31	9966.65	0.01	>99

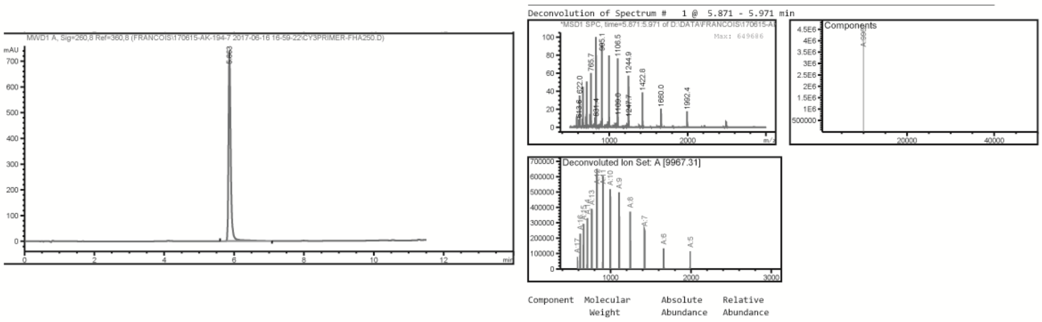

Name	Seq. 5'-3'	Chemistry	MW found (g/mol)	MW calc. (g/mol)	Δm (%)	UV purity (%)
**31**	AAATCGAGGGAGGGA$_{2'\text{-O-BHQ}}$G	DNA-PO BHQ label	5599.23	5598.15	0.02	>99

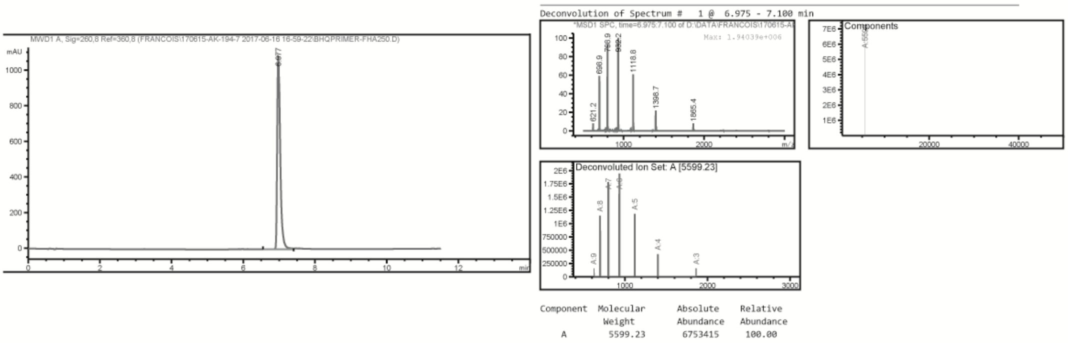

Name	Seq. 5'-3'	Chemistry	MW found (g/mol)	MW calc. (g/mol)	Δm (%)	UV purity (%)
**32**	BPS-CTCAGGCTGCTAACCACGT	DNA-PO 5' BPS label	5964.16	5966.06	0.03	>99

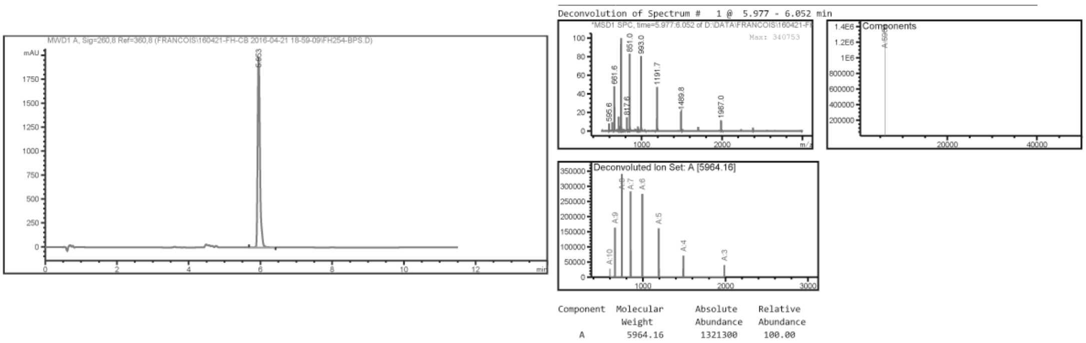

Figure S3. Analytics of in-vivo batches of ORN1, ORN$_{st}$, ORN$_{84}$ and ORN$_{ch}$

Oligonucleotide solutions were made in 0.3 M sodium acetate (ThermoFisher #AM9740) after RP-HPLC purification. Two rounds of ethanol precipitation were conducted to generate the compound as a sodium salt. Final dissolution was made in 0.9 % saline (Sigma-Aldrich #S8776), directly followed by a sterile filtration through 0.2 µm filter. An aliquot was sampled under sterile conditions to determine product concentration by A_{260} measurement and purity by LC-MS (Agilent 1200/6130 system) equipped with a Waters Acquity OST C-18 column.

ORN1

MW calc. (g/mol)	8770.60
MW found (g/mol)	8772.72
Δm (%)	0.02
UV purity (%)	>99

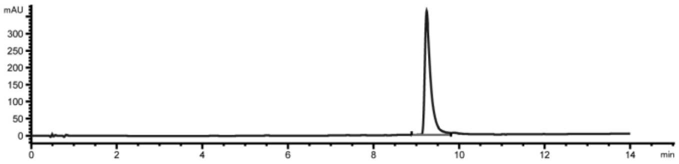

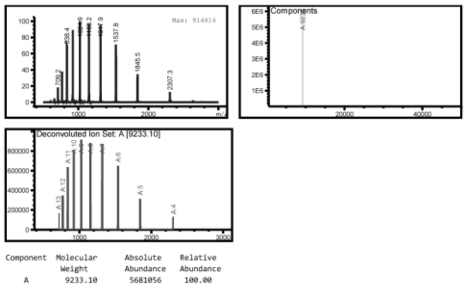

ORN^{84}

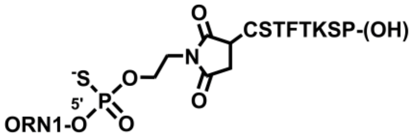

MW calc. (g/mol)	9859.15
MW found (g/mol)	9860.50
Δm (%)	0.01
UV purity (%)	>99

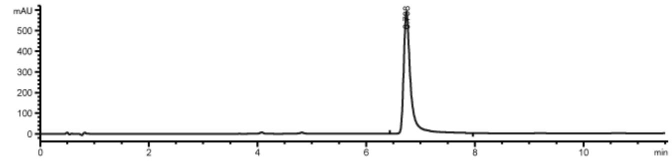

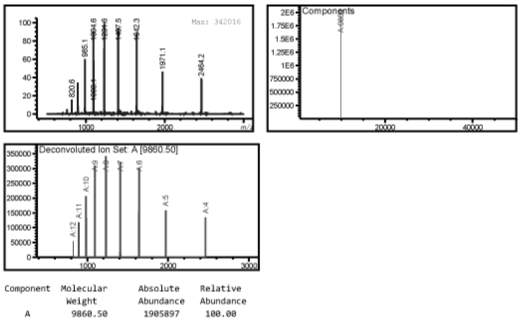

ORNst

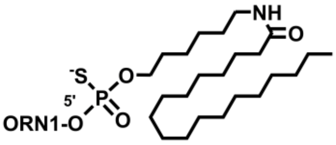

MW calc. (g/mol)	9233.32
MW found (g/mol)	9233.10
Δm (%)	0.01
UV purity (%)	>99

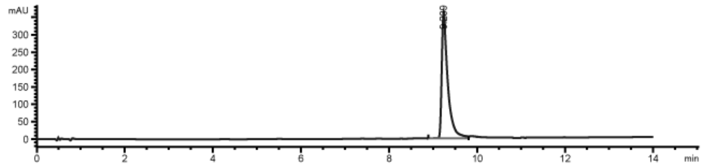

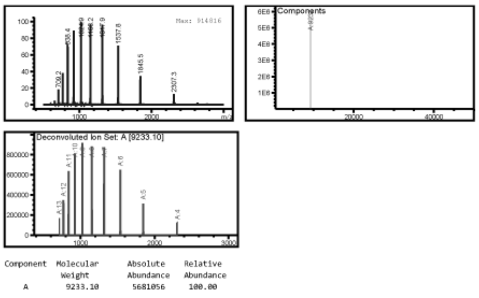

ORN^{ch}

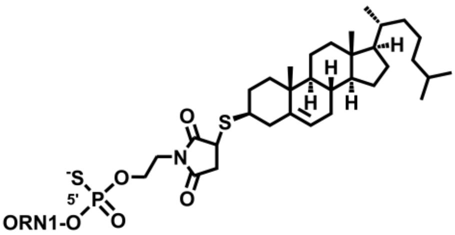

MW calc. (g/mol)	9392.42
MW found (g/mol)	9393.54<br>+ Na ion
∆m (%)	0.01
UV purity (%)	>99

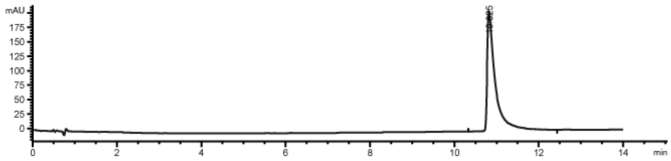

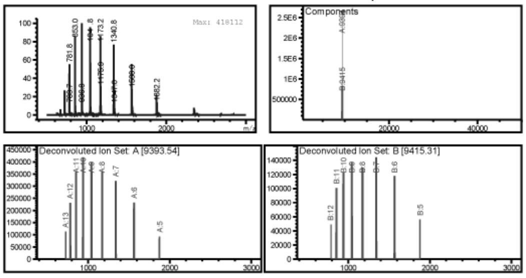

Component	Molecular Weight	Absolute Abundance	Relative Abundance
A	9393.54	2492164	100.00
B	9415.31	834157	33.47

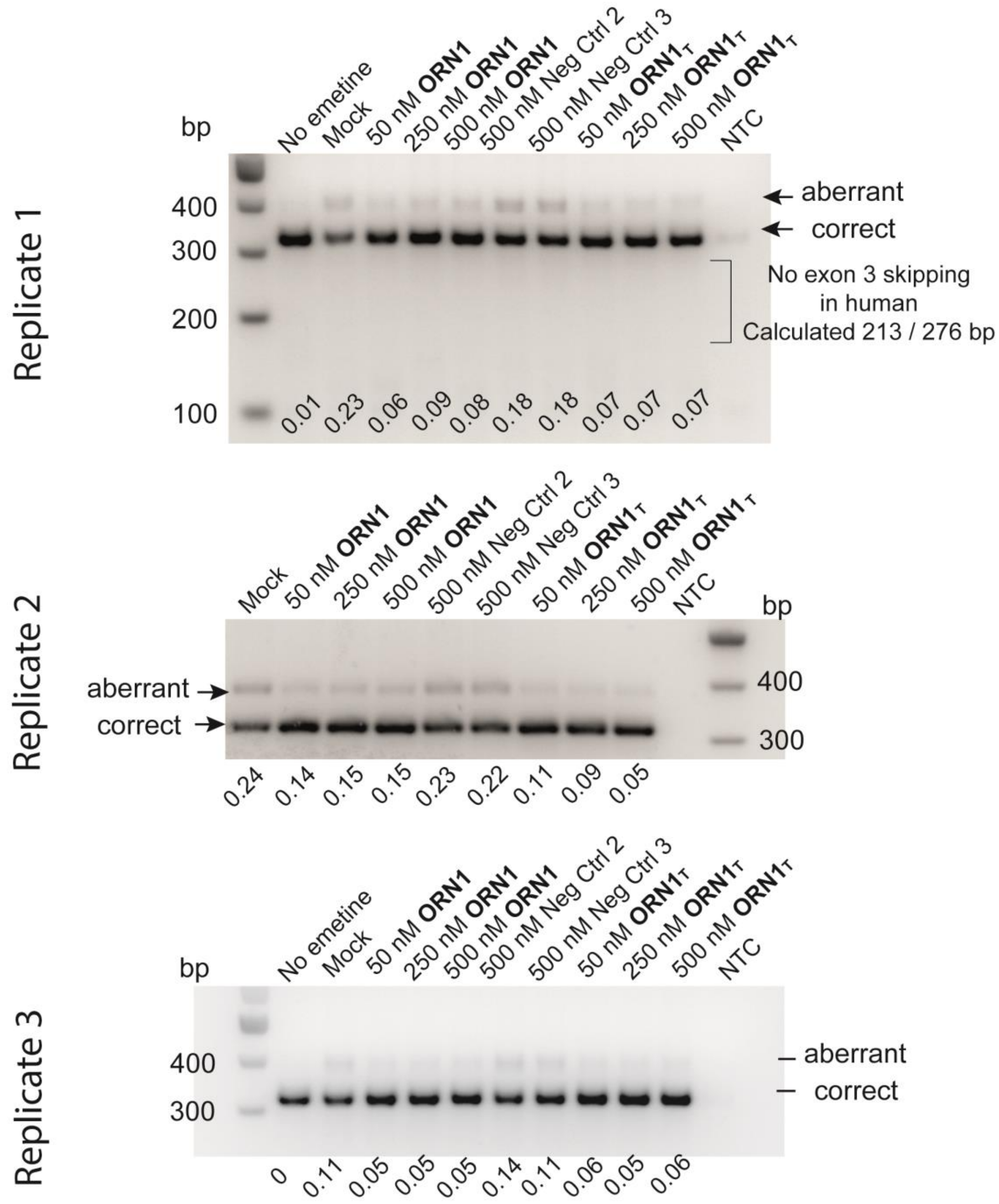

Figure S4. Gel replicates for *FECH* endogenous splicing correction in K562 cells.

K562 cells were were plated at 200.000 cells / well in a 24-well plate in 0.5 ml IMDE containing 10 % fetal bovine serum and 4 mM glutamine. Oligonucleotides were transfected using Lipofectamine™ 2000. 24 hours prior to lysis and RNA extraction, emetine was added in each desired well at a final concentration of 3 µM. RNA extraction was carried out using Qiagen RNEasy kit according to manufacturer's instructions. cDNA was generated using PrimeScript RT kit (Takara #RR037A). PCR primers for amplification of human *FECH* transcripts were used. PCR products were loaded on a 2% agarose gel and bands quantified using ImageJ software. Ratios were expressed as: intensity of the aberrant amplicon band / (intensities of aberrant + correct bands). As shown for Replicate 1, no exon 3 skipping is observed over PCR amplification. Exon 3-skipped species would have a size of 213 nt (correct) and 276 nt (aberrantly spliced).

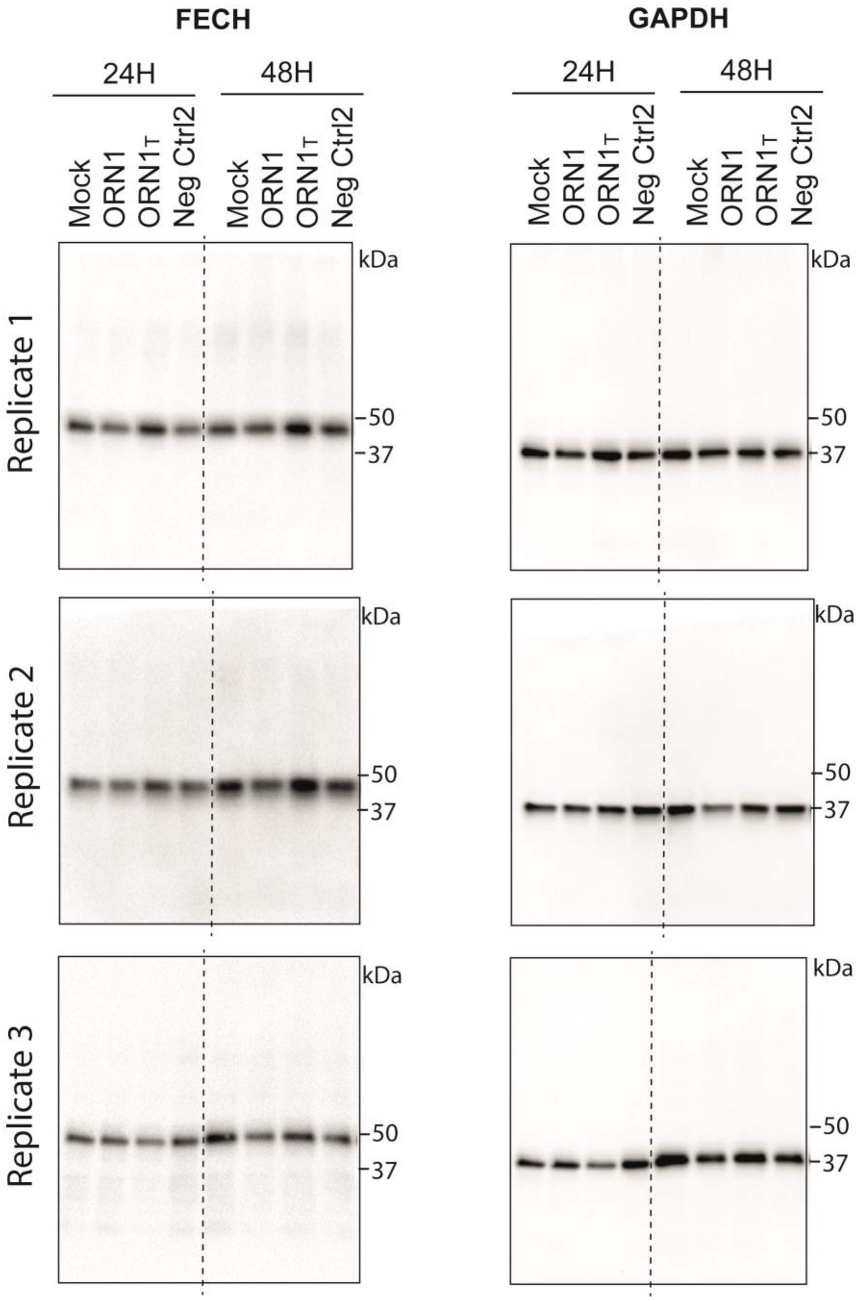

Figure S5. Western Blot replicates for FECH endogenous splicing correction in K562 cells.

K562 cells were were plated at 100.000 cells / well in a 24-well plate in 1 ml IMDE containing 10 % fetal bovine serum and 4 mM glutamine. Oligonucleotides were transfected using Lipofectamine™ 2000. Cells were lysed 24 or 48 h after treatment. Protein concentration was assessed with the Pierce BCA protein assay kit (ThermoFisher Scientific). Samples were loaded onto 4–20% pre-cast TGX gels (BioRad) for electrophoresis. The transfer of protein onto PVDF membranes (Roche) was carried out for 90 min at 25 W. Membranes were blocked with 3% BSA and were incubated with the primary antibody overnight at 4 °C. Membranes were washed with 0.05% PBS-T and reacted with the secondary antibody for 1 h in 5% milk. Binding was detected with the ECL Prime reagent (GE Life Sciences) and quantified using the ImageJ software. Graphical output and statistical analysis (Anova) were generated with GraphPad Prism software.

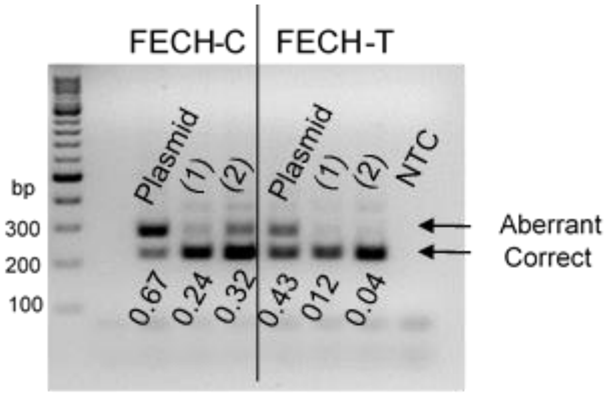

Figure S6. Aberrant splicing generated by the *FECH* c.315-48T and C minigenes

Agarose gel showing relative amounts of aberrant and correct transcripts from treatment (50 nM) of COS-7 cells expressing a FECH-C or a FECH-T minigene with -45-63 2'-OMe phosphorothioate SSOs complementary to the FECH-C (**1**) or the FECH-T (**2**) variants. Aberrant transcripts were stabilized with 3 μM emetine treatment. Data shows that both the C and T variants generate the aberrant transcript.

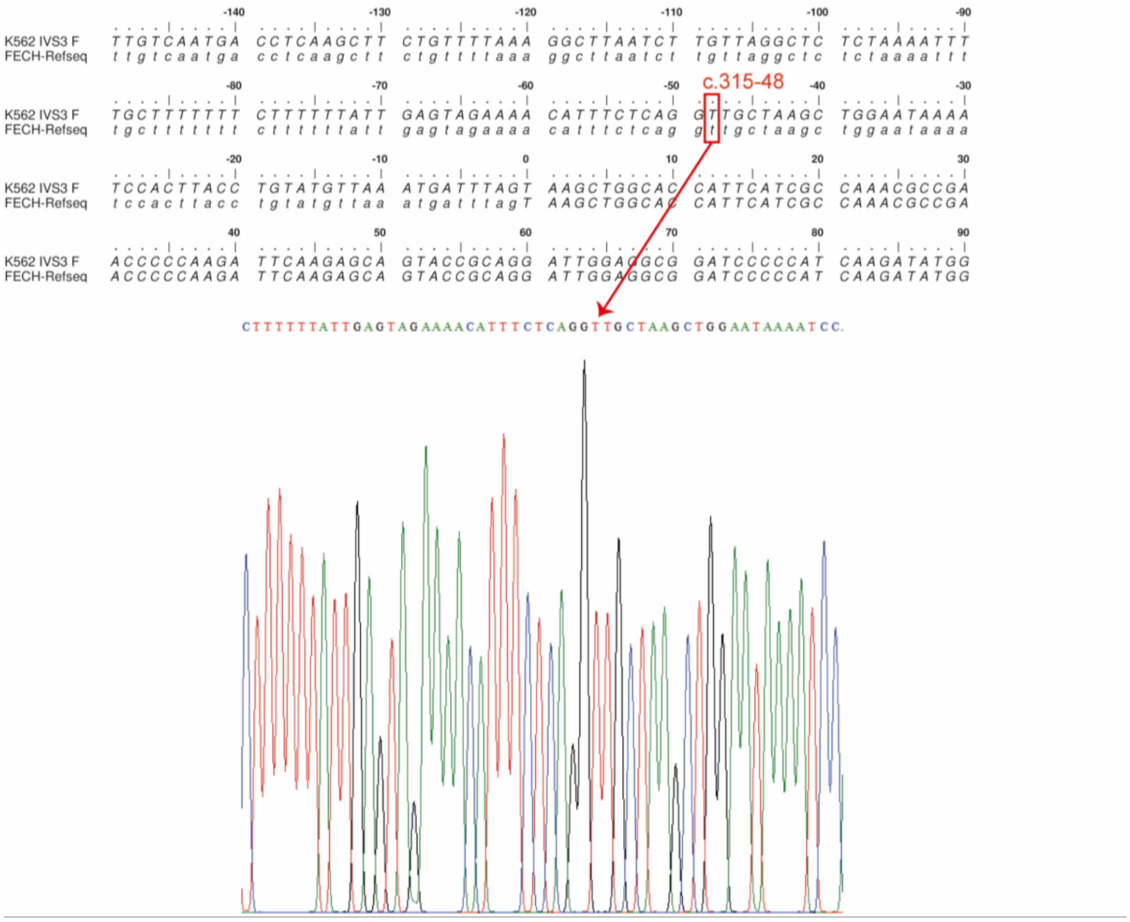

Figure S7. Status of the c.315-48 polymorphism in K562 cells: alignment of K562 gDNA sequencing data with *FECH* Human Reference Sequence (GRCh38.p13)

Crude genomic DNA was extracted using the KAPA Mouse Genotyping Kit (Merck #KK7301) as per manufacturer's instructions and was used as template for PCR amplification of the

genomic region containing the c.315-48 polymorphism using primers : Forward 5’-CTG TTG TGA ATG TGT AAA CTT AAC AC-3’; Reverse 5’-GAC AAT TCA TCC AGC AGC TTC-3’. A PCR mix containing 0.3 µl FW primer (10 µM stock), 0.3 µl RV primer (10 µM stock), 0.3 µl dNTPs mix (Promega #U1515), 2 µl 10X PCR buffer (Roche #12032902001), 0.2 µl Fast Taq DNA Polymerase (Roche #12032902001) and 14.9 µl ultrapure water was prepared and mixed with 2 µl of genomic DNA extract. PCR program: 95°C, 10min; 33 cycles (95°C, 30s – 53°C, 30s – 72°C, 45s); 68°C, 5 min. PCR products were loaded on a 2% agarose gel for analysis. PCR products were purified for DNA sequencing using Qiaquick PCR purification kit (Qiagen # 28104). Sanger sequencing was carried out using the same primers as for PCR amplification (Microsynth DNA sequencing services).

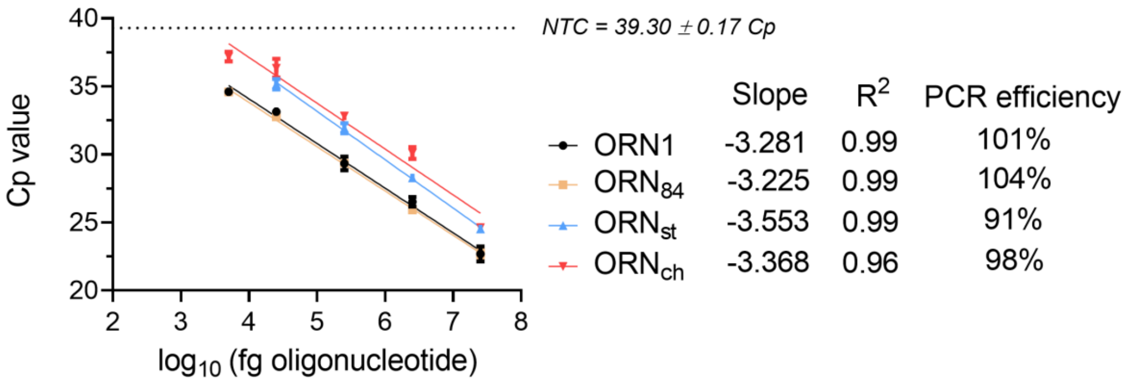

Figure S8. Calibration curves of oligonucleotide conjugates in kidney lysate
ORN1 and its conjugated forms were spiked in kidney lysate diluted at 1/750 in nanopure water and serially diluted. Chemical ligation was run for 1 h at 33°C. qPCR was run on a LC480 device (Roche) with technical triplicates for each datapoint. Cp values were calculated by the onboard software. Slopes and R^2 values were calculated with GraphPad Prism v7. software. PCR efficiency values were calculated with the web qPCR Efficiency calculator from ThermoFisher Scientific.

In vivo metabolism study in mouse tissues: mass spectrometry data (Figures S9 to S12)

Grinded tissues were suspended in 10 volumes/weight OTX Lysis Buffer, briefly needle-sonicated and centrifuged for 30 s at 14000 rpm. Supernatants were collected for oligonucleotide clean-up and directly processed with the Clarity® OTX Extraction kit (Phenomenex #KS0-9253), according to manufacturer's procedure for tissues with two modifications: formulation of all buffers with ammonium acetate instead of sodium phosphate; and dissolution of the final eluate in 100 µl of TE buffer pH 8.0 instead of water after evaporation to dryness. Optimization of the protocol with liver samples from mice treated with cholesteryl conjugate **ORN^{ch}** comprised a proteinase K pre-treatment of the tissue to ensure recovery of the oligonucleotide (this pre-treatment was used for all **ORN1^{ch}** -related treatments). Samples were analysed by LC-MS (Agilent 1200/6130 system) equipped with a Waters Acquity OST C-18 column with a gradient of MeOH in 0.4 mM HFIP, 15 mM trimethylamine; flow rate: 0.3 ml/min. Deconvolution of the mass spectra returned molecular weights from which the structures of metabolites were inferred.

Figure S9. MS data of ORN1 in tissues

Kidney: oligonucleotide peak at 5.448 min

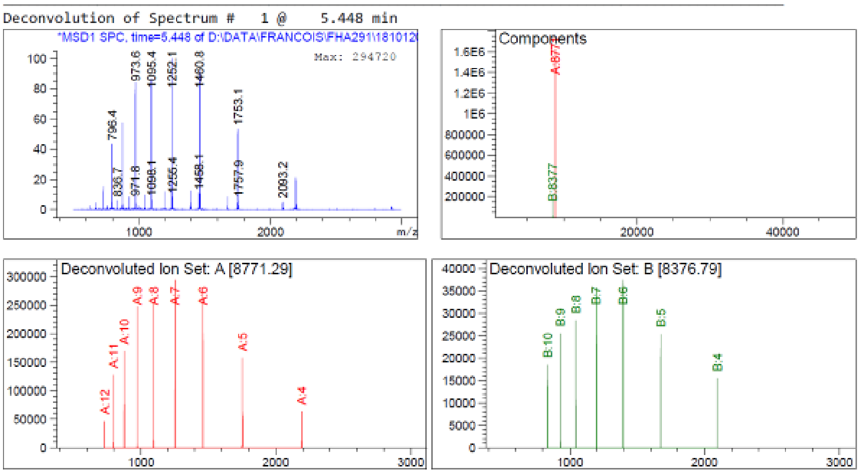

Component	Molecular Weight	Absolute Abundance	Relative Abundance
A	8771.29	1618850	100.00
B	8376.79	181305	11.20

*** End of Report ***

Liver: Oligonucleotide peak at 5.448 min; n-1 metabolite manually integrated (black arrows)

Deconvolution of Spectrum # 1 @ 5.157 - 5.257 min

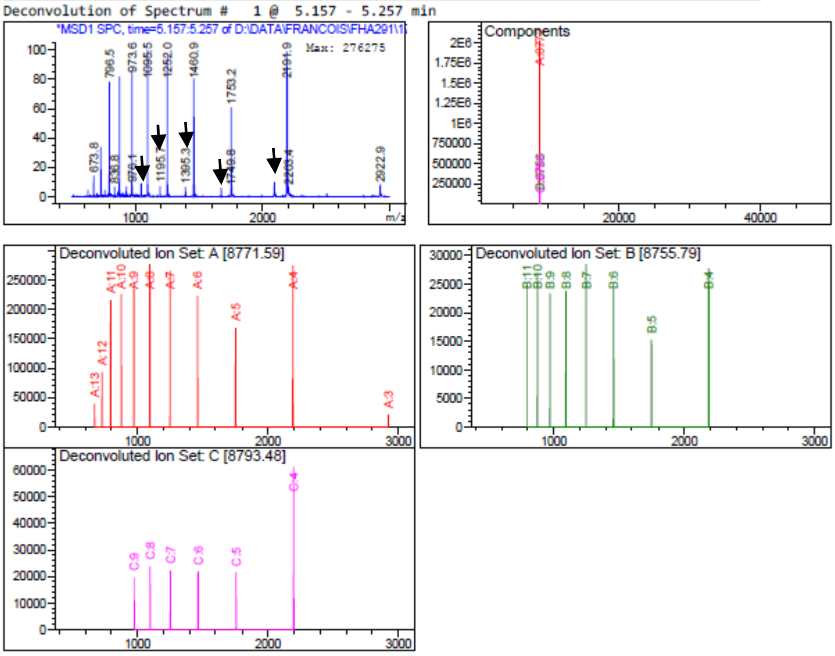

Component	Molecular Weight	Absolute Abundance	Relative Abundance
A	8771.59	2015827	100.00
B	8755.79	189327	9.39
C	8793.48	168602	8.36

*** End of Report ***

Spleen: oligonucleotide peak at 5.556 min; n-1 metabolite manually integrated (black arrows)

Deconvolution of Spectrum # 1 @ 5.556 min

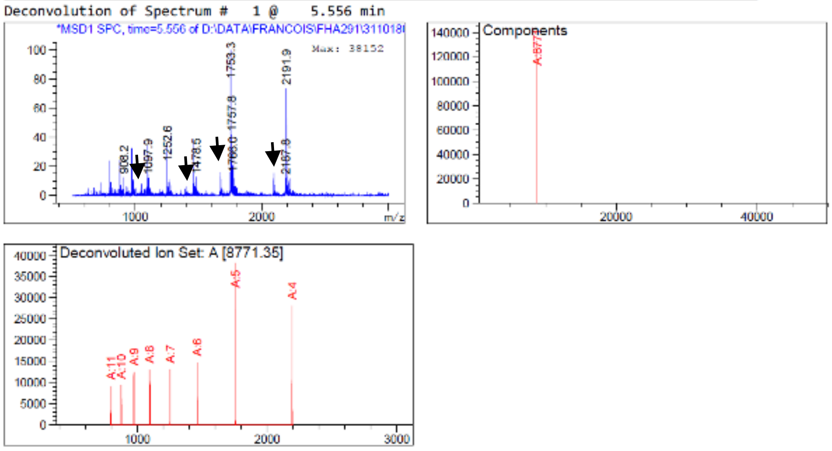

Component	Molecular Weight	Absolute Abundance	Relative Abundance
A	8771.35	132671	100.00

*** End of Report ***

Brain: no oligonucleotide detected within sensitivity limits of our equipment

Lung: oligonucleotide peak at 5.581 min

Deconvolution of Spectrum # 1 @ 5.581 min

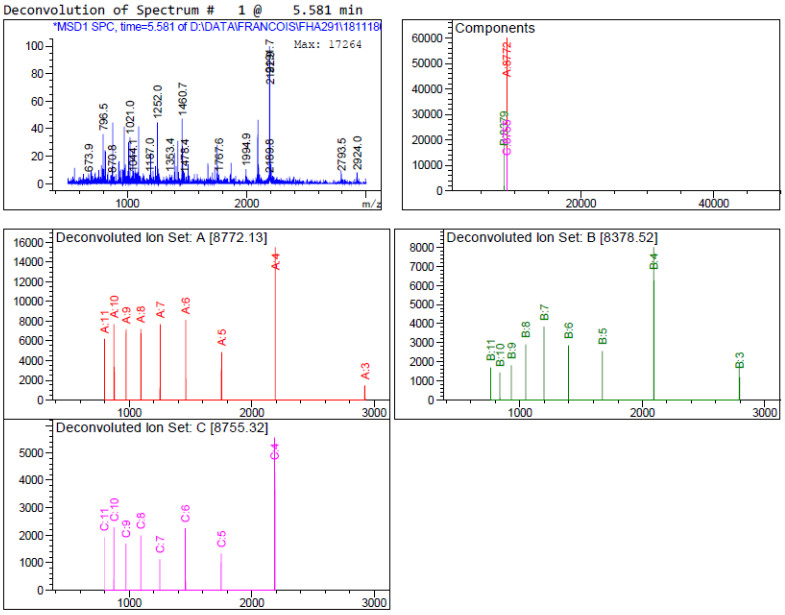

Component	Molecular Weight	Absolute Abundance	Relative Abundance
A	8772.13	59914	100.00
B	8378.52	20764	34.66
C	8755.32	16170	26.99

*** End of Report ***

Bone marrow: oligonucleotide peak at 5.581 min

Deconvolution of Spectrum # 1 @ 5.581 min

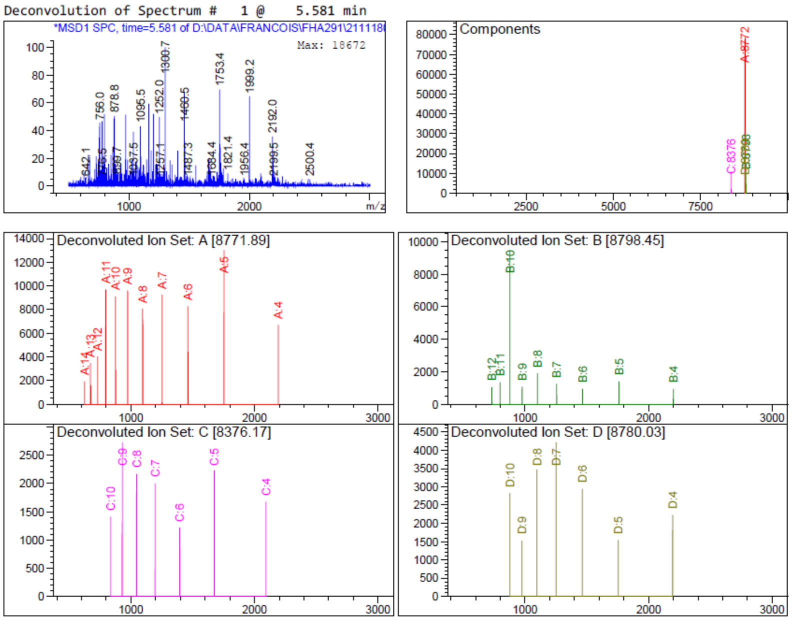

Component	Molecular Weight	Absolute Abundance	Relative Abundance
A	8771.89	77746	100.00
B	8798.45	14106	18.14
C	8376.17	11039	14.20
D	8780.03	10869	13.98

*** End of Report ***

Figure S10. MS data of ORN_{st} in tissues

Kidney: oligonucleotide peak at 5.456 min

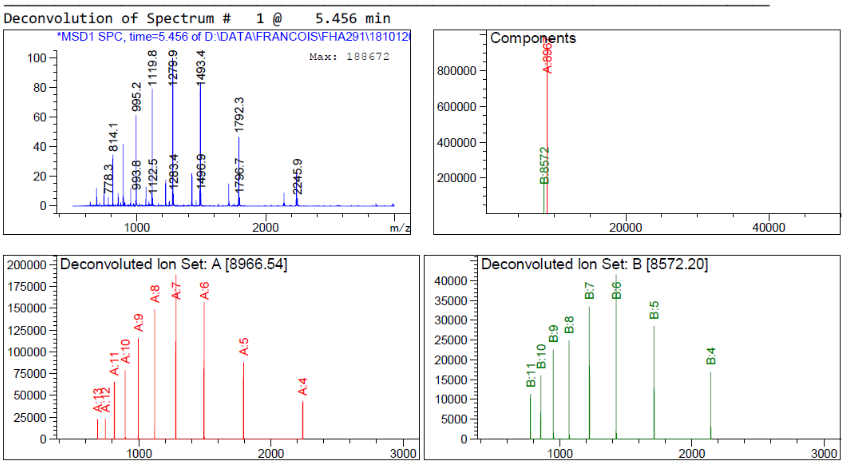

Component	Molecular Weight	Absolute Abundance	Relative Abundance
A	8966.54	922098	100.00
B	8572.20	189976	20.60

*** End of Report ***

Liver: two oligonucleotide peaks at 5.381 and 9.292 min

Peak at 5.381 min

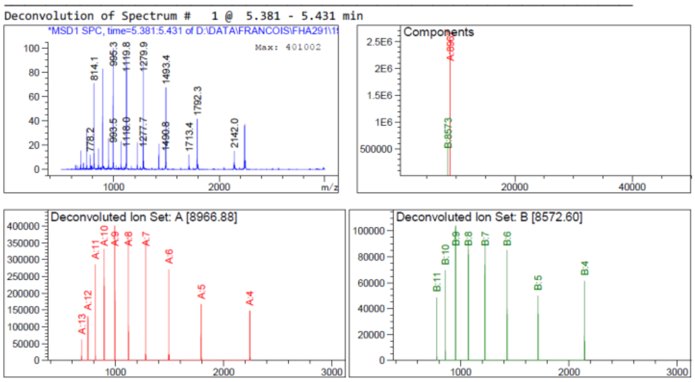

Component	Molecular Weight	Absolute Abundance	Relative Abundance
A	8966.88	2509402	100.00
B	8572.60	586390	23.37

Peak at 9.292 min (next page)

Deconvolution of Spectrum # 1 @ 9.292 min

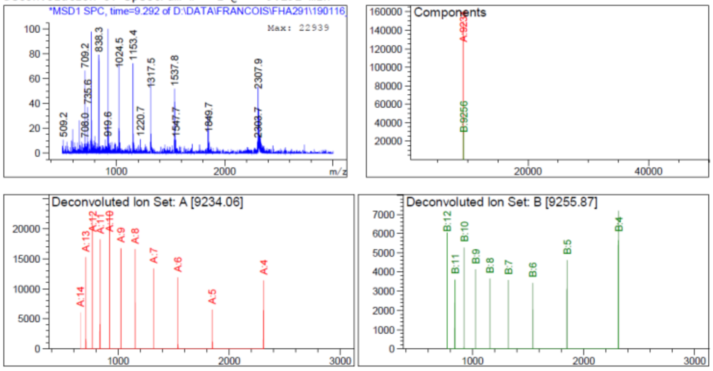

Component	Molecular Weight	Absolute Abundance	Relative Abundance
A	9234.06	149277	100.00
B	9255.87	34066	22.82

Lung: oligonucleotide peak at 5.506 min

Deconvolution of Spectrum # 1 @ 5.506 min

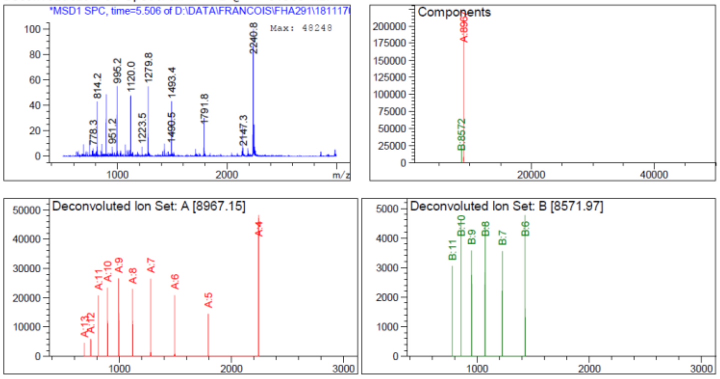

Component	Molecular Weight	Absolute Abundance	Relative Abundance
A	8967.15	206787	100.00
B	8571.97	20236	9.79

*** End of Report ***

Brain: oligonucleotide peak at 5.481 min

Deconvolution of Spectrum # 1 @ 5.481 - 5.680 min

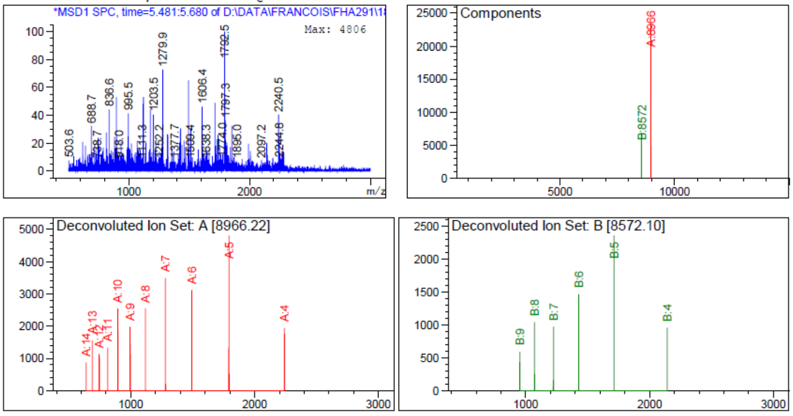

Component	Molecular Weight	Absolute Abundance	Relative Abundance
A	8966.22	23466	100.00
B	8572.10	6763	28.82

Bone marrow: oligonucleotide peak at 5.597 min

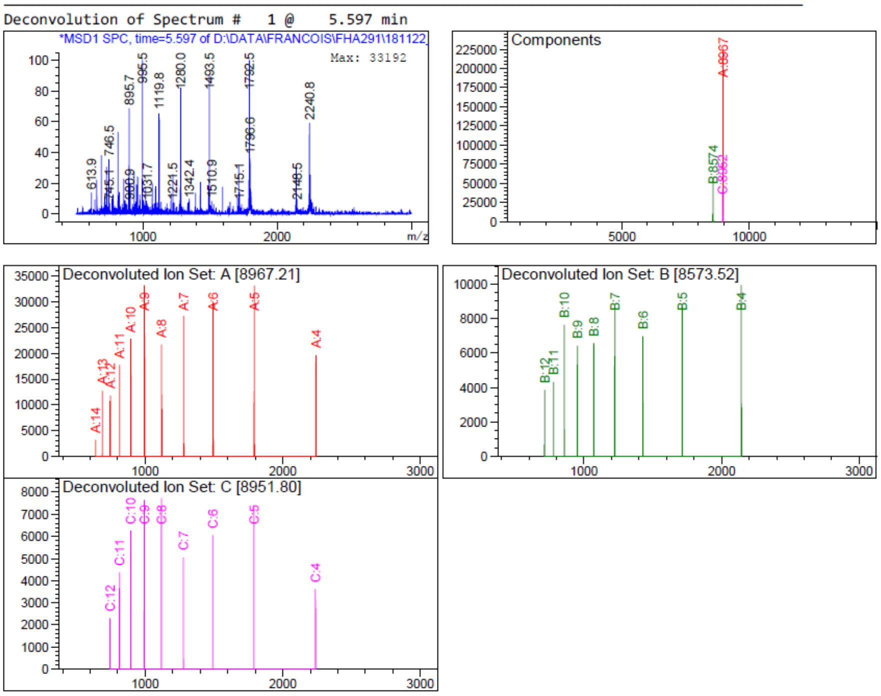

Component	Molecular Weight	Absolute Abundance	Relative Abundance
A	8967.21	222624	100.00
B	8573.52	57352	25.76
C	8951.80	42249	18.98

Figure S11. MS data of ORN_{84} in tissues

Kidney: oligonucleotide peak at 5.456 min, n-1 metabolite manually integrated (black arrows)

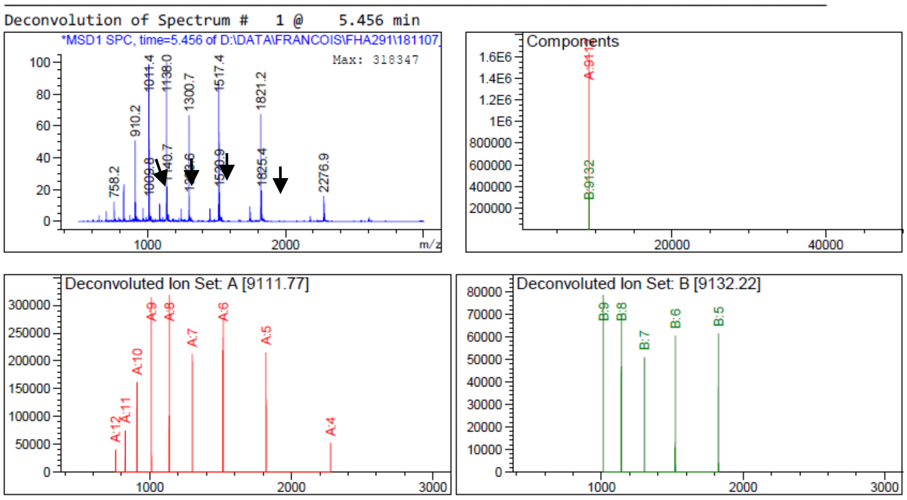

Component	Molecular Weight	Absolute Abundance	Relative Abundance
A	9111.77	1632938	100.00
B	9132.22	314986	19.29

*** End of Report ***

Liver: oligonucleotide peak at 5.331 min

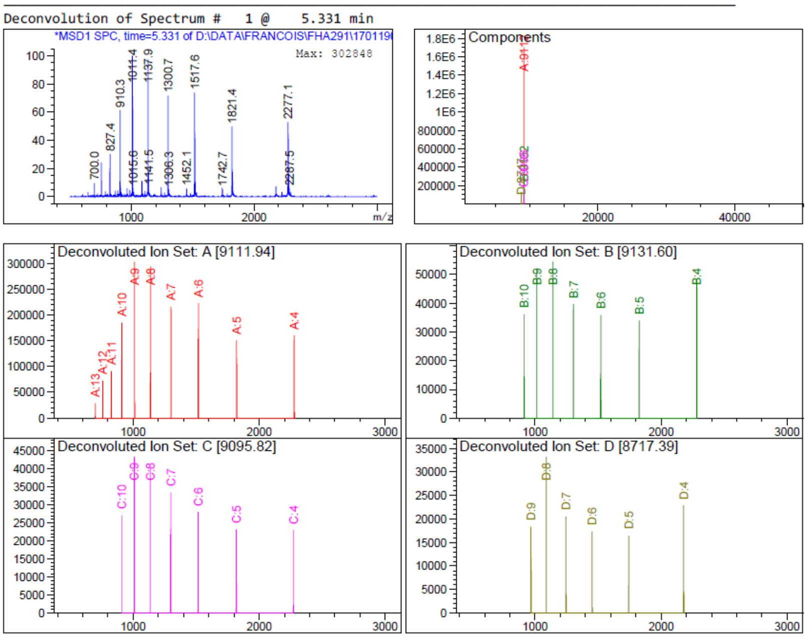

Component	Molecular Weight	Absolute Abundance	Relative Abundance
A	9111.94	1703340	100.00
B	9131.60	285055	16.74
C	9095.82	207578	12.19
D	8717.39	120787	7.09

*** End of Report ***

Lung: oligonucleotide peak at 5.431 min

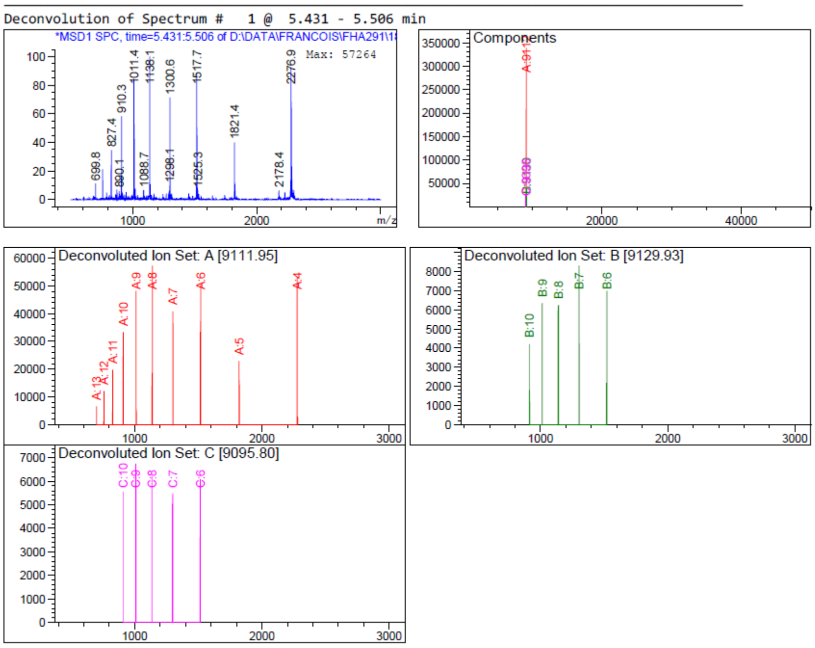

Component	Molecular Weight	Absolute Abundance	Relative Abundance
A	9111.95	338329	100.00
B	9129.93	31450	9.30
C	9095.80	27493	8.13

*** End of Report ***

Bone marrow: oligonucleotide peak at 5.506 min

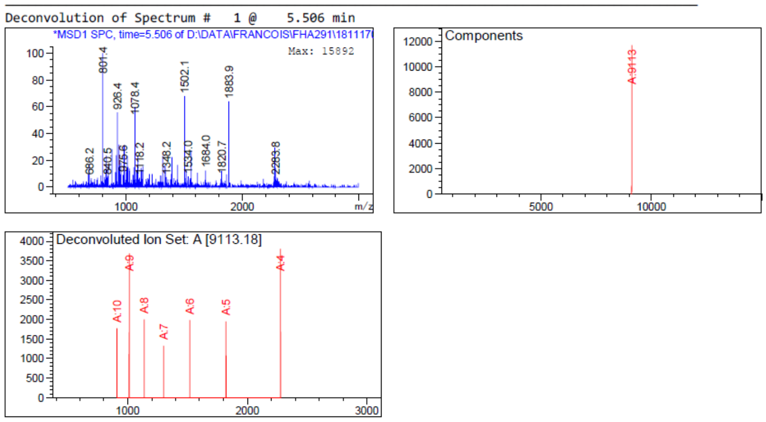

Component	Molecular Weight	Absolute Abundance	Relative Abundance
A	9113.18	11703	100.00

Brain: no oligonucleotide detected within sensitivity limits of our equipment

Spleen: oligonucleotide peak at 5.431 min

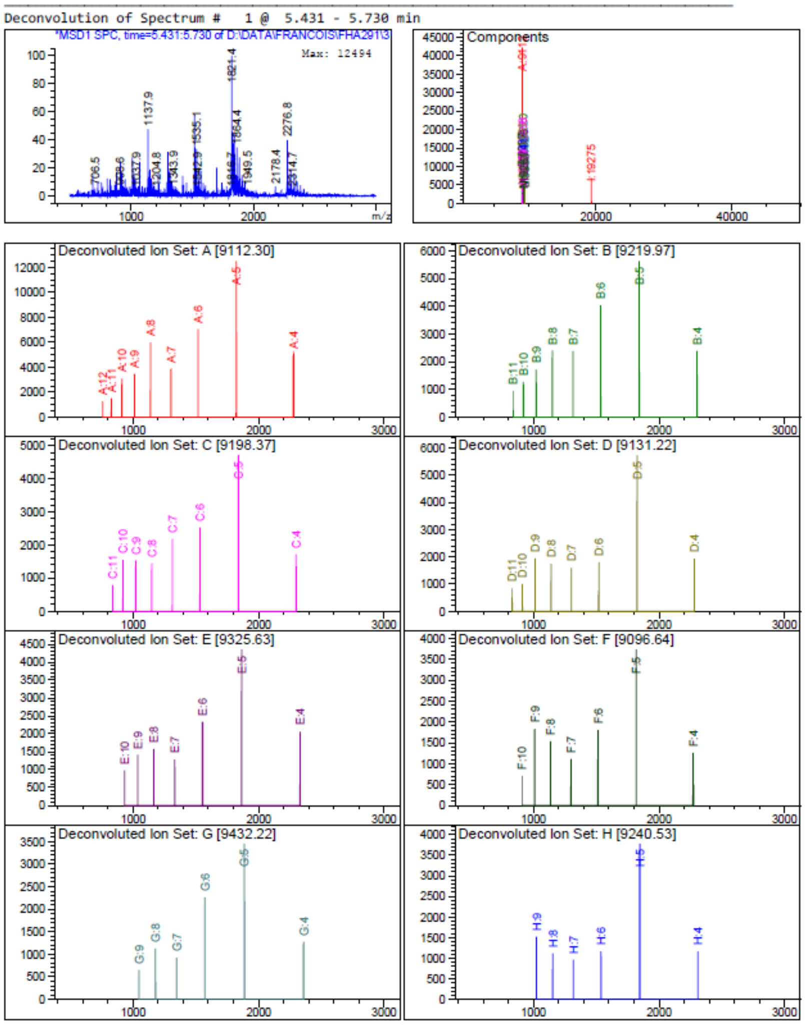

Component	Molecular Weight	Absolute Abundance	Relative Abundance
A	9112.30	42205	100.00
B	9219.97	17292	40.97
C	9198.37	16014	37.94
D	9131.22	14088	33.38
E	9325.63	12336	29.23
F	9096.64	11456	27.14
G	9432.22	9391	22.25
H	9240.53	9088	21.53

Figure S12. MS data of ORN$_{ch}$ in tissues

Kidney: two oligonucleotide peaks at 5.356 min (two main metabolites analyzed) and 11.334 min

Peak at 5.356 min

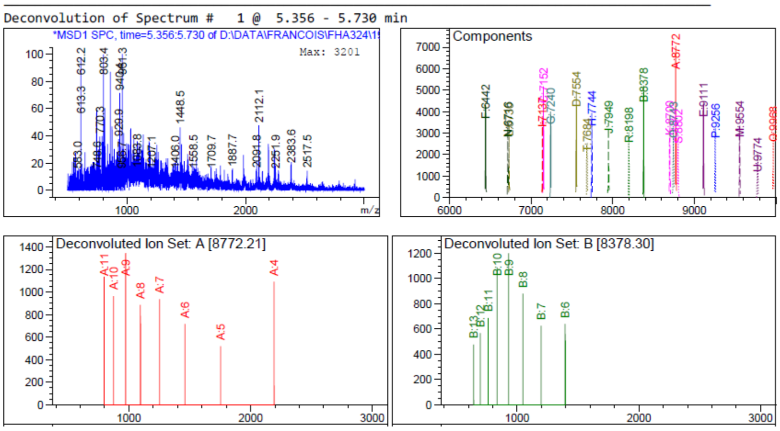

Component	Molecular Weight	Absolute Abundance	Relative Abundance
A	8772.21	7060	100.00
B	8378.30	5210	73.80

Peak at 11.34 min

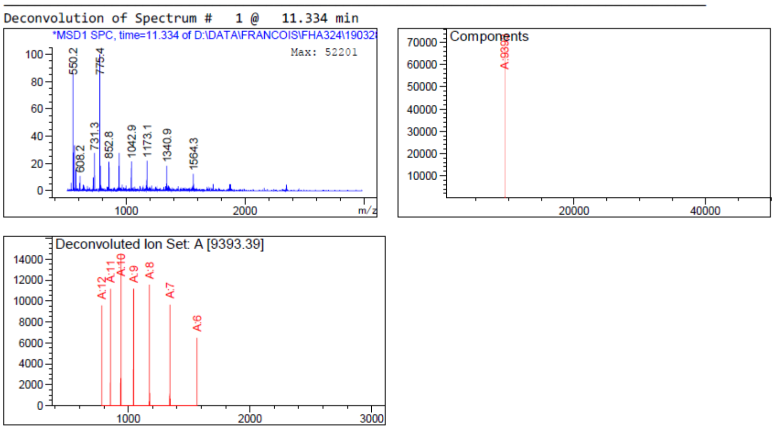

Component	Molecular Weight	Absolute Abundance	Relative Abundance
A	9393.39	68361	100.00

*** End of Report ***

Liver: two oligonucleotide peaks at 5.406 min (two main metabolites analyzed) and 10.886 min

Peak at 5.406 min

Deconvolution of Spectrum # 1 @ 5.406 min

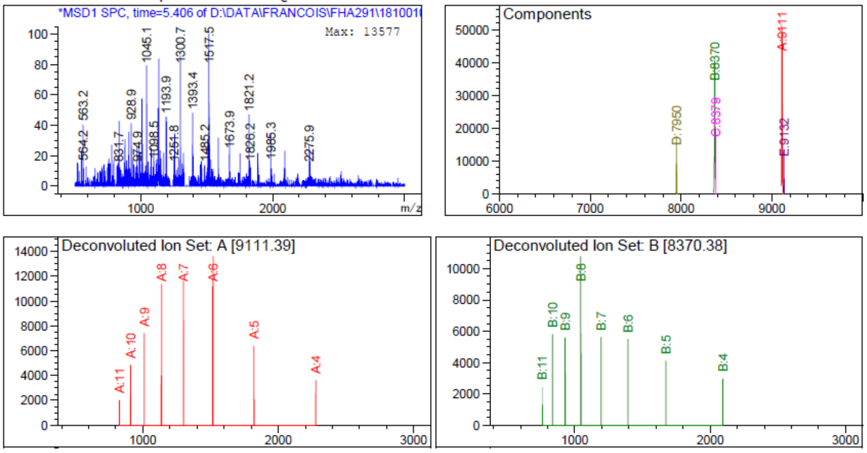

Component	Molecular Weight	Absolute Abundance	Relative Abundance
A	9111.39	51395	100.00
B	8370.38	40597	78.99

Peak at 10.886 min

Deconvolution of Spectrum # 1 @ 10.886 min

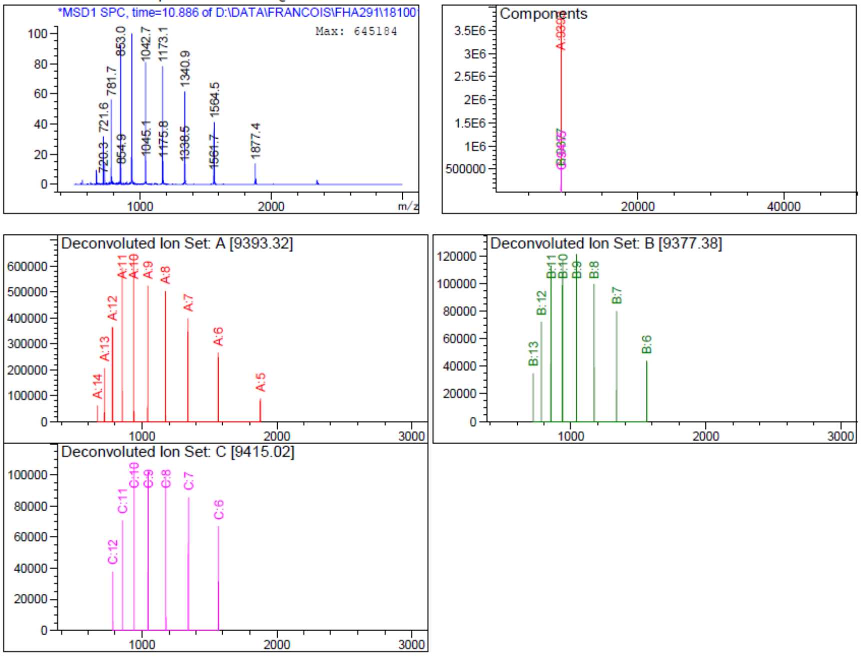

Component	Molecular Weight	Absolute Abundance	Relative Abundance
A	9393.32	3625731	100.00
B	9377.38	662765	18.28
C	9415.02	566194	15.62

*** End of Report ***

Spleen: oligonucleotide peak at 11.409 min

Deconvolution of Spectrum # 1 @ 11.409 - 11.833 min

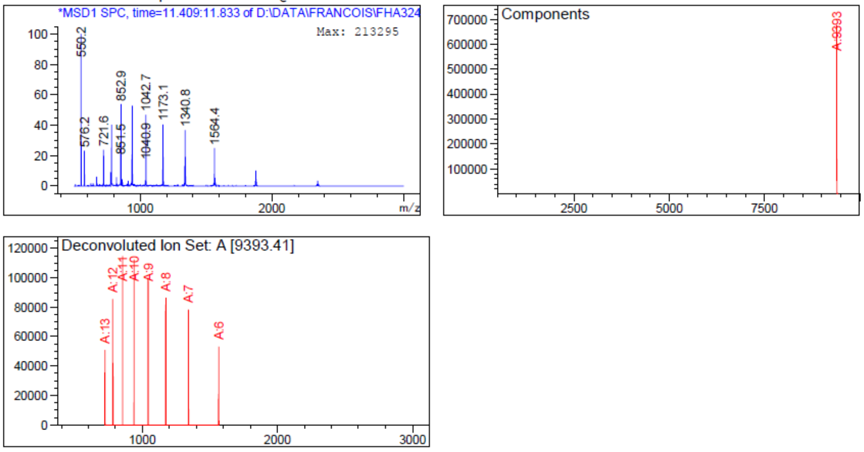

Component	Molecular Weight	Absolute Abundance	Relative Abundance
A	9393.41	676649	100.00

*** End of Report ***

Lung: oligonucleotide peak at 11.882 min

Deconvolution of Spectrum # 1 @ 11.882 min

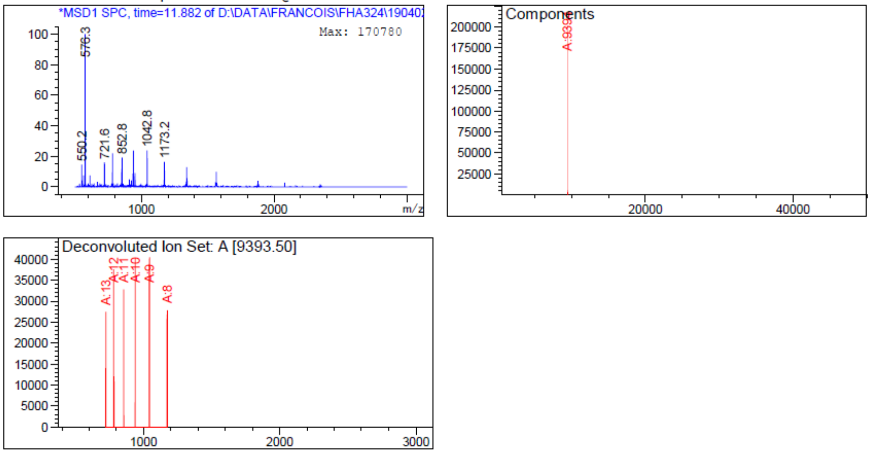

Component	Molecular Weight	Absolute Abundance	Relative Abundance
A	9393.50	202387	100.00

*** End of Report ***

Brain: no oligonucleotide detected within sensitivity limits of our equipment

Bone marrow: two oligonucleotide peaks at 5.507 min and 11.884 min (next page)

Peak at 5.507 min

Deconvolution of Spectrum # 1 @ 5.507 min

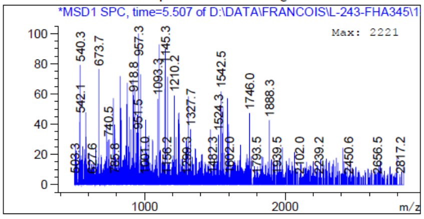

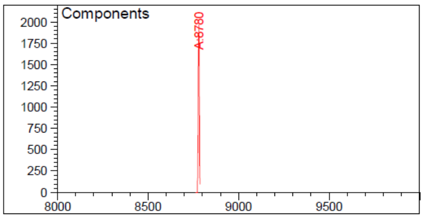

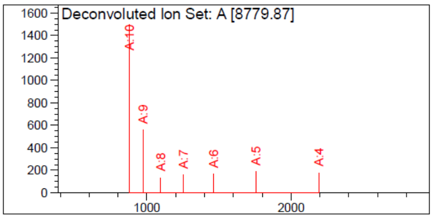

Component	Molecular Weight	Absolute Abundance	Relative Abundance
A	8779.87	1966	100.00

*** End of Report ***

Peak at 11.884 min

Deconvolution of Spectrum # 1 @ 11.884 min

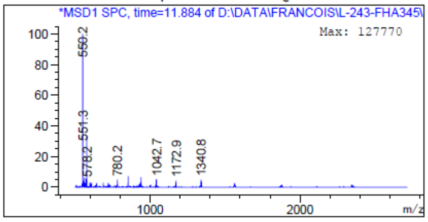

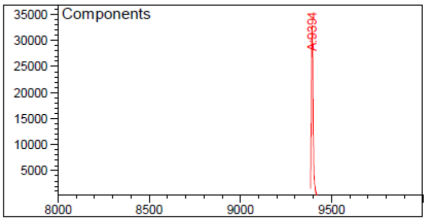

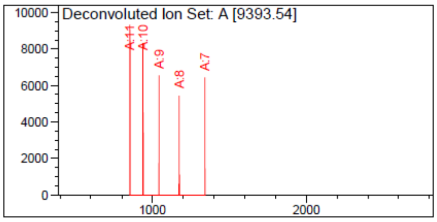

Component	Molecular Weight	Absolute Abundance	Relative Abundance
A	9393.54	32952	100.00

*** End of Report ***

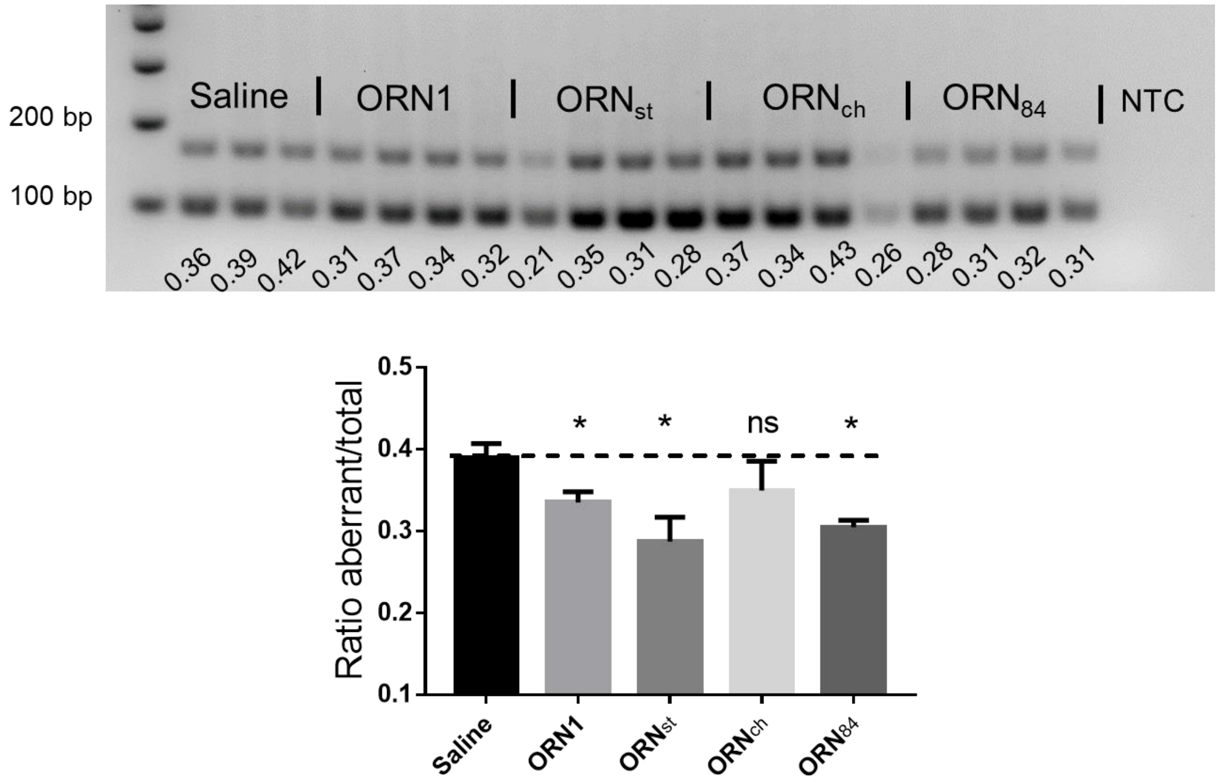

Figure S13. Splicing correction generated by ORN1, ORN_{st}, ORN_{ch} and ORN_{84} in liver of *Emi/wt* mice.

RNA was extracted from liver tissue as described in the Methods section. For semi-quantitative RT-PCR; PCR primers specific for amplification of *Emi* exon 3-exon 4 transcripts were used: Forward 5'- GAG ACC TCA TGA CAC TTC-3', Reverse 5'- GGG GAT CCG CCT CCA ATC -3'. A PCR mix containing 1.25 µl FW primer (10 µM stock), 1.25 µl RV primer (10 µM stock), 0.125 µl dNTPs mix (Promega #U1515), 0.25 µl 5X Q5 Polymerase Buffer (NEB #M0491), 0.25 µl Q5 DNA Polymerase (NEB #M0491) and 15.75 µl ultrapure water was prepared and mixed with 1 µl of cDNA solution. PCR program: 98°C, 30s; 33 cycles (98°C, 10s – 59°C, 20s – 72°C, 10s); 72°C, 2 min. PCR products were loaded on a 2% agarose gel and bands quantified using ImageJ software. Ratios were expressed as: intensity of the aberrant amplicon band / (intensities of aberrant + correct bands). Statistical analysis (multiple *t*-tests) were conducted with GraphPad Prism v7. Software (* = p <0.05). Data are shown on the graph as average ratio values ± s.e.m.

Supplementary tables

Table S1. Library of 10-nt ORNs for minigene screening

Oligonucleotides were produced and characterized as described earlier. Sequences are fully modified MOE-PS chemistry (^{m}C refers to 5-methyl cytidine).

Name	Seq. 5'-3'	Target	MW calc. (g/mol)	MW found (g/mol)	Δm (%)	UV purity (%)
**33**	A^{m}CT^{m}CAATAAA	-75-66	3916.6	3917.6	0.02	93
**34**	T^{m}CTA^{m}CT^{m}CAAT	-72-63	3888.5	3889.2	0.02	99
**35**	TTTT^{m}CTA^{m}CT^{m}C	-69-60	3870.4	3871.6	0.03	98
**36**	ATGTTTT^{m}CTA	-66-57	3906.5	3905.3	0.03	97
**37**	GAAATGTTTT	-63-54	3941.5	3940.8	0.02	>99
**38**	TGAGAAATGT	-60-51	3975.6	3974.6	0.03	98
**39**	T^{m}CTA^{m}CT^{m}CAAT	-57-48	3973.6	3975.1	0.04	97
**40**	G^{m}CAG^{m}C^{m}CTGAG	-54-45	3979.6	3979.9	0.01	>99
**41**	TTAG^{m}CAG^{m}C^{m}CT	-51-42	3929.5	3929.2	0.01	>99
**42**	AG^{m}CTTAG^{m}CAG	-48-39	3964.6	3965.6	0.02	>99
**43**	T^{m}C^{m}CAG^{m}CTTAG	-45-36	3929.5	3929.8	0.01	>99
**44 Neg Ctrl 4**	TAT^{m}CATT^{m}CA^{m}C	Scrambled	3888.5	3888.9	0.01	>99

Supplementary methods

Deprotection and purification conditions for lead oligonucleotide ORN1

The CPG was suspended in fresh 25 % ammonia (Sigma Aldrich) and shaken at 45°C for 22 hours. The CPG was filtered, washed with 50 % (aq) EtOH and the liquid fractions were evaporated to dryness prior to dissolution in ultrapure water. After brief centrifugation, the crude DMT-ON oligonucleotide was purified by RP-HPLC (Agilent) with a reverse phase column XBridge OST C18 with a gradient of acetonitrile in 0.1 M aqueous triethylammonium acetate buffer, pH 8. Fractions containing the product were evaporated to dryness and redissolved for detritylation in 50 % (aq) acetic acid. Detritylation was allowed to run under moderate shaking for 1 hour at RT prior to evaporation to dryness and a second round of RP-HPLC purification with the same system. Fractions containing pure product were combined and evaporated to dryness prior to redissolution in ultrapure water.

Conjugation on support, deprotection and purification of stearic acid conjugate ORN_{st}

The amino C6 modifier (Glen Research #10-1906) was prepared at 0.1M; synthesis was conducted with same parameters as above. The 5' MMT group was deprotected by flushing 3 x 10 ml of Deblock solution through the CPG. The CPG was washed with 2 x 10 ml of acetonitrile and allowed to react with a solution of 160 mg stearic NHS ester and 80 µl DIPEA in 8 ml DMF for 30 min. After washing with 2 x 10 ml of acetonitrile, standard deprotection and purification protocols were applied as for naked compound **ORN1**, however only one round of RP-HPLC was conducted.

Deprotection of maleimide-protected form of ORN1 and retro Diels Alder uncaging for conjugation to thiol ligands

The 5'-Maleimide modifier (Glen Research #10-1938) was prepared at 0.1M; synthesis was conducted with same parameters as above. The use of the 5'-maleimide modifier was so far successful with Pac-DNA amidites (2,3) and protocol fine-tuning was required to achieve in the case of MOE oligonucleotides in one hand full deprotection of the nucleobases and cleavage from support, while keeping in the other integrity of the 5' protected maleimide modification. With final conditions, the CPG was suspended in fresh 25 % ammonia (Sigma Aldrich) and shaken at 35°C for 18 hours. For the same reaction time, t temperatures ≥ 40°C led to unacceptable maleimide degradation; temperatures ≤ 30°C to uncomplete nucleobase deprotection. Purification protocol was done as described for **ORN1** but only with one round of RP-HPLC. The 5'-protected maleimide oligonucleotide was analyzed by LC-MS and stored at -20°C at this stage in water; only required amounts were engaged whenever needed for uncaging of the 5'-maleimide and conjugation.

Retro-Diels Alder reaction was conducted using a modified protocol from the literature (3): the 5'-maleimide intermediate was prepared at a concentration of 100 µM in water and microwave-irradiated for 90 minutes at 90°C, followed by immediate concentration in a Speedvac until ≈ 90 % volume

reduction was obtained. Evaporation to dryness was avoided. LC-MS injection was routinely carried out to control quality of the retro Diels-Alder deprotection prior to conjugation.

Thiocholesteryl conjugate preparation $\mathbf{ORN_{ch}}$ from the uncaged maleimide intermediate

A solution of thiocholesterol (Sigma-Aldrich #136115) was prepared in THF at 10 mg/ml and was added to the concentrated solution of maleimide intermediate in water to reach 10 molar eq. of thiocholesterol. The final solvent ratio was adjusted if necessary to get a ratio THF/H_2O of 3:1. The mixture was degassed by argon bubbling and microwave-irradiated for 25 min at 30°C. RP-HPLC purification was then carried out as for compound **ORN1**, but with one round of RP-HPLC only.

CD84-binding peptide conjugate preparation $\mathbf{ORN_{84}}$ from the uncaged maleimide intermediate

A solution of peptide (Genscript) was prepared in water at 10 mg/ml and was added to the concentrated solution of maleimide intermediate in water to reach 7 molar eq. of peptide. The reaction was buffered with TEAA at pH 7 and allowed to run for 30 minutes at RT. Product $\mathbf{ORN^{84}}$ was purified by RP-HPLC on the same system as for **ORN1** but with milder conditions: 4 ml/min, 40°C and a gradient of acetonitrile in 0.1 M TEAA buffer pH 6.8.

References

1. Pradère, U., Brunschweiger, A., Gebert, L.F.R., Lucic, M., Roos, M. and Hall, J. (2013) Chemical Synthesis of Mono- and Bis-Labeled Pre-MicroRNAs. *Angewandte Chemie International Edition*, **52**, 12028-12032.
2. Sánchez, A., Pedroso, E. and Grandas, A. (2011) Maleimide-Dimethylfuran exo Adducts: Effective Maleimide Protection in the Synthesis of Oligonucleotide Conjugates. *Organic Letters*, **13**, 4364-4367.
3. Sánchez, A., Pedroso, E. and Grandas, A. (2012) Easy introduction of maleimides at different positions of oligonucleotide chains for conjugation purposes. *Organic & Biomolecular Chemistry*, **10**, 8478-8483.
